# Toward Long‐Term Reliable Human‐Machine Interaction: A Flexible, Breathable, and Self‐Powered Pressure Sensor System With Firefighting Validation

**DOI:** 10.1002/advs.75660

**Published:** 2026-05-14

**Authors:** Qilong Zhang, Zhao Yao, Jiaxu Liu, Yuxuan Hou, Zhongtao Zhang, Jingwei Xue, Leonid Chernogor, Nam Young Kim, Eun Seong Kim, Yuanyue Li, Yang Li

**Affiliations:** ^1^ Shandong Key Laboratory of Micro–nano Packaging and System Integration College of Electronic and Information Qingdao University Qingdao China; ^2^ Tumor Precise Intervention and Translational Medicine Laboratory The Affiliated Taian City Central Hospital of Qingdao University Taian China; ^3^ Shandong Provincial Key Medical and Health Laboratory of Microenvironment Response Biomedical Materials Taian City Central Hospital Taian China; ^4^ RF Bio Center Department of Electronic Engineering Kwangwoon University Seoul South Korea; ^5^ Laboratory of Molecular Pathology and Cancer Genomics Department of Molecular Medicine and Biopharmaceutical Sciences Graduate School of Convergence Science and Technology Seoul National University Seoul South Korea; ^6^ Department of Medical and Digital Engineering College of Engineering Hanyang University Seoul South Korea; ^7^ School of Integrated Circuits Shandong University Jinan China

**Keywords:** fire trolley control system, hydrogel electrolyte, identity verification system, potentiometric pressure sensor, sensing performance

## Abstract

To overcome the continuous power problem for wearable applications, the self‐powered sensors have attracted much attention recently. The traditional piezoelectric and triboelectric sensors can only generate signals under dynamic force. Herein, a zinc–iodine based potentiometric sensor is proposed for simultaneous and dynamic force detection. The proposed sensor is based on a multilayer architecture consisting of a breathable nonwoven fabric acting as the substrate, a Zn‐plated laser‐induced graphene (LIG) anode, a LIG cathode coated with an iodine‐based composite ink, and a polyvinyl alcohol/zinc chloride hydrogel serving as the electrolyte. Such a design increases the open circuit voltage to 1.31 V, and provides stable sensing over an extended pressure range from 4 to 256 kPa. The fabricated sensor exhibits a rapid response time of 72 ms, excellent cycling stability (>98% retention after 5000 cycles), and superior wearing comfort. A gesture‐recognition glove integrated with five sensing units was developed and allowed for the correct recognition of gestures and wirelessly controlling a fire detection smart car that is equipped with temperature/gas sensors and camera, enabling real‐time remote monitoring in a complex environment. This work provides an innovative strategy for self‐powered and highly comfortable interfaces in emergency scenarios.

## Graphical abstract

This work develops a breathable potentiometric self‐powered pressure sensor with good wearability, using a non‐woven fabric substrate and hydrogel electrolyte. The sensor is integrated into a five‐channel smart glove to acquire reliable hand motion signals, which are processed by a CNN for identity verification and smart vehicle control in human‐machine interaction scenarios.

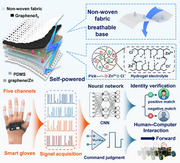

## 1 Introduction

With the wide applications of wearable electronics in the smart home, electronic skin, and health monitor, the development of self‐powered sensing technology is still moving toward higher reliability and comfortable wearing [1, 2, 3, 4, 5, 6, 7, 8, 9, 10, 11, 12, 13, 14]. For task like firefighting and emergency response in complex domains, it is imperative to have human‐machine interaction systems that can operate stably over a prolonged period of time, enabling real‐time on‐site information acquisition and precise remote device control‐factors directly critical to rescue efficiency and personnel safety [15]. To accommodate these extreme cases, a perfect pressure sensor would be able to respond in two modes: fast for impact and slow for pressure, while providing good breathability and biocompatibility for wearing comfort. There are clear shortcomings of the available solutions: despite being able to achieve a dual‐mode sensing with conventional capacitive or resistive sensors, their dependence on an external power supply, which limits their use over long periods at risky places [16, 17, 18, 19, 20, 21]. Commonly used self‐powered piezoelectric/triboelectric sensors are likewise self‐powered and require no external source, although they tend to struggle with static or quasi–static pressure signals in particular [22, 23].

Within the pathway of dual‐mode self‐powered sensing, two main technical routes currently exist. The first is a mixed approach consisting of piezoelectric/triboelectric coupled with capacitive/resistive elements. This method has the disadvantages of signal crosstalk, structural complexity, and high costs, limiting its practical application [24, 25, 26, 27, 28]. The second route involves potentiometric sensors based on redox reactions at the electrode–electrolyte interface, which merge self‐powering and dual‐mode detection within a simple structure, demonstrating unique potential. Nevertheless, several challenges remain for their practical implementation: the output voltage is generally low; most designs employ non‐breathable substrates, significantly impairing long‐term wearing comfort; and the use of liquid electrolytes introduces risks of leakage and evaporation, raising stability concerns [29, 30, 31, 32, 33, 34]. For instance, the potentiometric–triboelectric hybrid sensor developed by Wu et al. achieved dual‐mode response but still exhibited an output voltage below 1 V [35, 36]. Dai et al. developed a zinc‐ion hybrid supercapacitor‐based mechanical‐electrochemical conversion device capable of self‐powered sensing and adjustable energy supply. However, its encapsulation with PET and Kapton tape may compromise skin breathability in wearable applications [37]. Kim et al.’s functional sponge sensor based on liquid electrolyte was limited by encapsulation reliability and output stability [31]. Thus, though continuous advances in electrochemical interface engineering, there remains a challenge to achieve wearing comfort (e.g., insufficient breathability) while maintaining high output voltage and long‐term stability. It is still a major bottleneck for the deployment of this technology to long‐duration and high‐reliability applications.

To overcome these issues, in this work we develop a potentiometric pressure sensor based upon a zinc–iodine (Zn–I_2_) electrochemical system. The proposed sensor employs a medical nonwoven fabric (NF) as the substrate, fabricating the electrode system via laser‐induced graphene (LIG) with screen‐printing processes, and a polyvinyl alcohol (PVA)/zinc chloride (ZnCl_2_) hydrogel as the solid electrolyte [38, 39]. This design offers both high ionic conductivity and interface stability as well as good breathability and wearing comfort. Its operating mechanism is based on pressure modulated the electrode–electrolyte contact area, which is used to directly regulate the Zn–I_2_ redox reaction to generate an electrical signal positively correlated with the applied pressure. The sensor achieves a high open‐circuit voltage of 1.31 V, sufficient to directly power microelectronic devices such as light emitting diodes (LEDs), and demonstrates outstanding dual‐mode response performance for both dynamic and static pressures. Furthermore, the sensor was integrated into a firefighting glove to construct an intelligent interactive system capable of real‐time gesture recognition, remote control of a detection vehicle, and simultaneous reception of multi‐modal environmental data including temperature, gas concentration, and video feedback. It allows for a reliable remote control as well as real‐time situational awareness in hazardous rescue scenarios. Experimental results also confirm the high responsiveness and operational stability of the system in practical applications, offering a new and useful wearable solution for emergency response in high‐risk environments.

## 2 Result and Discussion

### 2.1 Sensor Design and Fabrication

**FIGURE 1:**
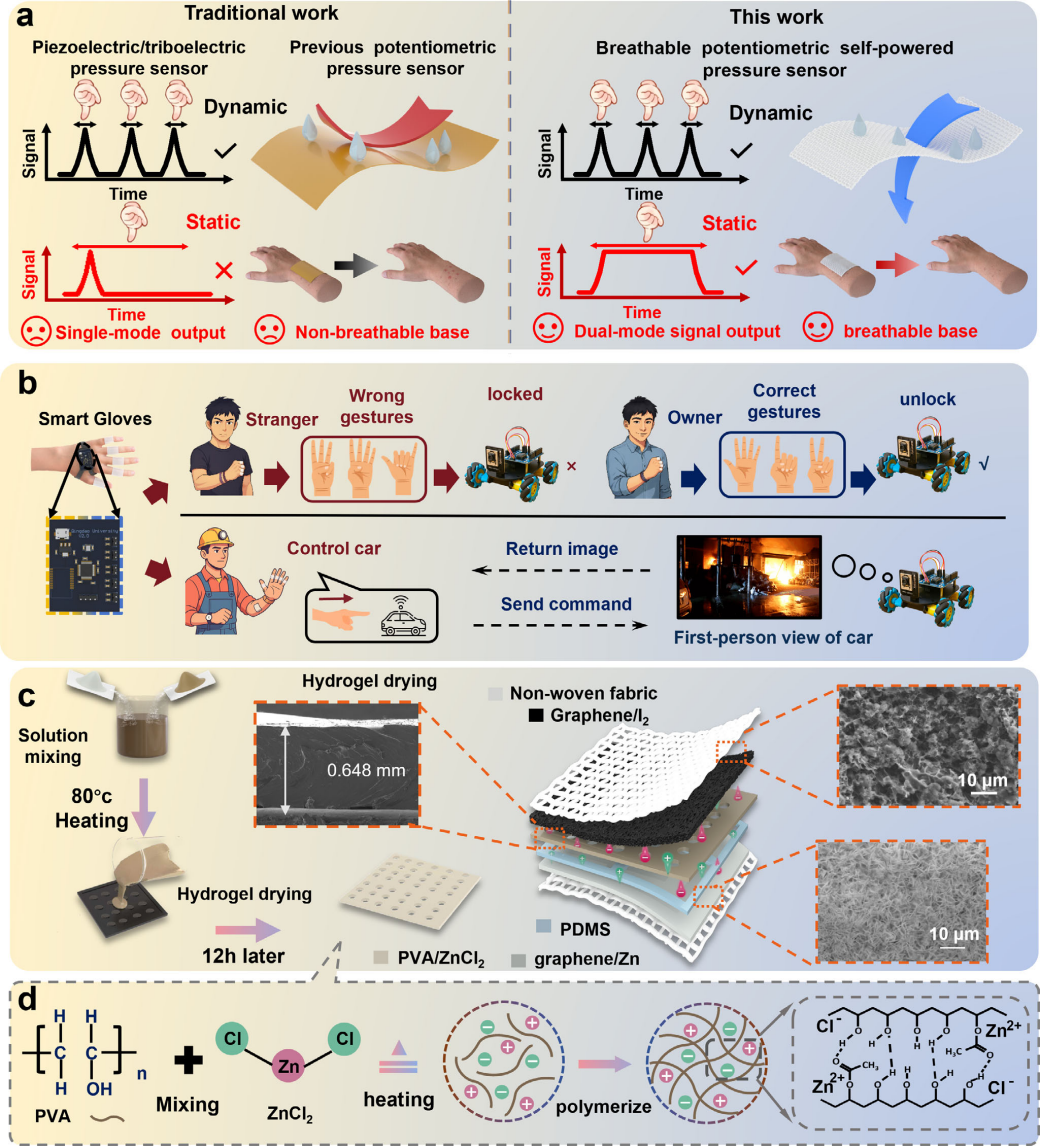
Sensor design and fabrication. (a) Comparative analysis of conventional self‐powered pressure sensors and the flexible, breathable, potentiometric pressure sensor developed in this study. (b) Schematic diagram of a smart glove integrated with the sensors for identity recognition and vehicle control system. (c) Fabrication process of the multilayer‐structured sensor. (d) Cross‐linking process of the PVA/ZnCl_2_ hydrogel.

In this work, a self‐powered potentiometric flexible pressure sensor was presented. It can not only respond to both static and dynamic pressure, but also provide excellent breathability (Figure 1a). Based on the above sensor, an integrated sensing system was developed and incorporated into a smart glove, combining two key functionalities: identity authentication and remote operation in hazardous conditions (Figure 1b). In terms of security authentication, the system recognizes specific gesture sequences via a machine learning algorithm to enable vehicle unlocking. For remote operation, the system allows real‐time monitoring of environmental parameters, such as temperature, gas concentration, and first‐person‐view video, enabling reliable remote control of vehicles in dangerous scenarios, such as fire emergencies.

The sensor was fabricated using a multilayer structural design (Figure 1c; Figure S1). The top iodine electrode was prepared by LIG film (Figure S2), followed by its transfer to a non‐woven fabric substrate (Figure S3). A functional ink composed of I_2_, CNTs, acetylene black, and glycerol was then coated onto it. The bottom zinc electrode was fabricated by electroplating zinc onto the surface of LIG. It was observed that the electrical conductivity of LIG decreased after being transferred to the non‐woven fabric but significantly improved during the subsequent zinc electroplating process (Figure S4). The Scanning electron microscopy (SEM) characterization confirmed the successful loading of zinc and iodine ions on the LIG surface. The middle layer consisted of a hydrogel electrolyte with a thickness of 0.64 mm, formed by dehydration‐induced crosslinking of a PVA and ZnCl_2_ solution at room temperature(Figure 1d); this thickness was determined to be optimal after testing 0.2, 0.64, and 1.5 mm, since thinner gels (0.2 mm) suffer from insufficient mechanical strength, rapid water loss, and low/unstable signals, while thicker gels (1.5 mm) compromise wearability and degrade sensing performance (Figure S5), while the isolation layer was fabricated from polydimethylsiloxane (PDMS) by spin coating (1500 rpm, 15 s, thickness 2 mm) and mechanical drilling (hole diameter 3 mm) to provide physical spacing between the electrodes without impeding ionic conductivity. A systematic comparison of pore sizes (2, 3, and 4 mm) indicates that a pore size of 2 mm restricts the contact between the electrolyte and the electrode as well as ion transport, leading to a low or non—existent output voltage. A pore size of 4 mm results in premature contact saturation and poor signal stability. In contrast, a pore size of 3 mm optimally balances effective contact, ion transport, and structural integrity (Figure S6).

### 2.2 Sensor Characterization and Biocompatibility Assessment

**FIGURE 2:**
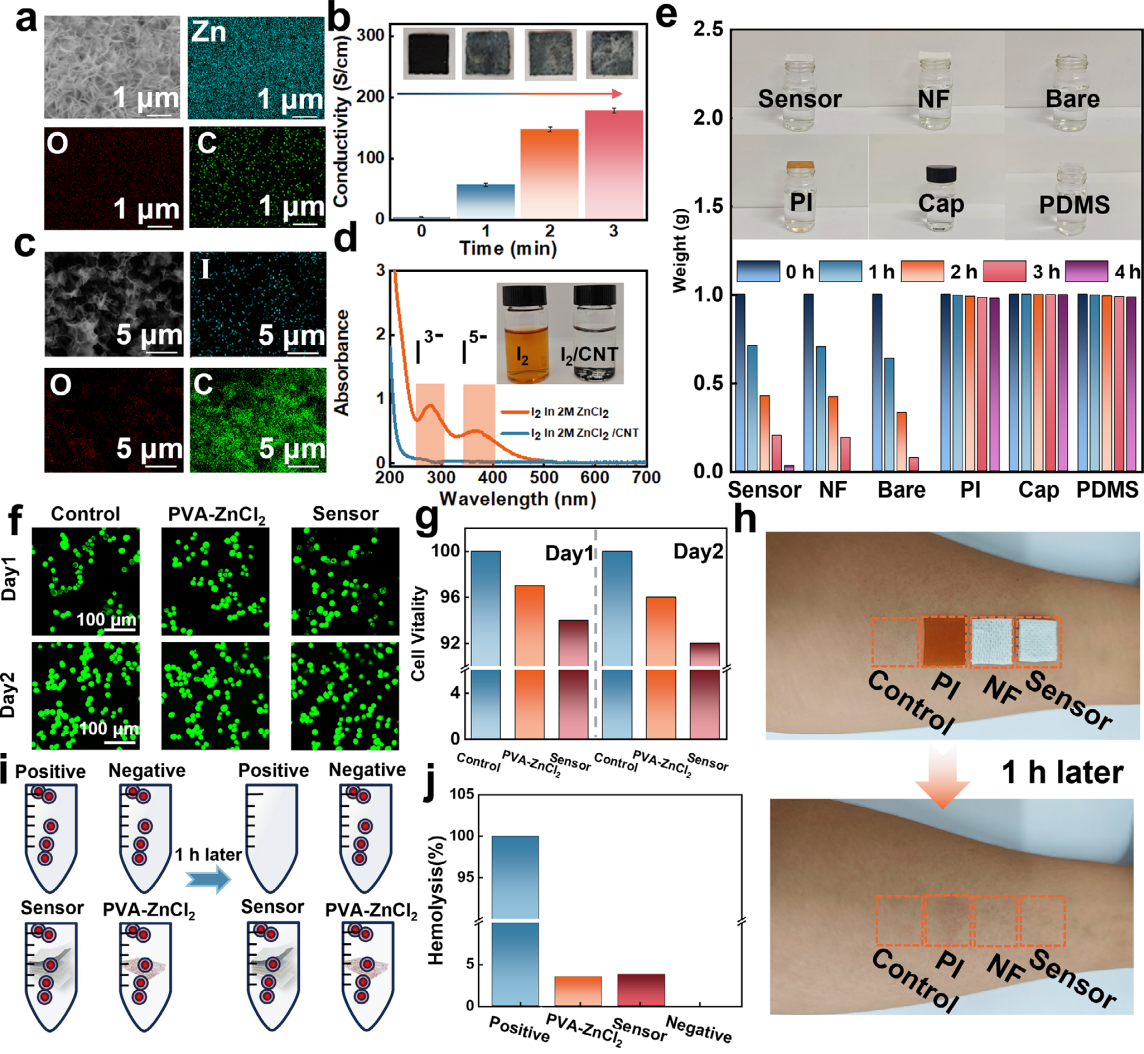
Sensor characterization and biocompatibility assessment. (a) EDS elemental mapping showing the distribution of iodine on the electrode surface. (b) Influence of electroplating time on zinc layer thickness and electrode conductivity. (c) EDS elemental mapping showing the distribution of zinc on the electrode surface. (d) Adsorption capability of CNTs for iodide ions, characterized by UV–vis absorption spectroscopy. (e) Breathability evaluation after heating at 100°C for 4 h. (f) Fluorescence images of live/dead stained cells after 24 h of culture for different groups. (g) Quantitative cell viability of different groups after 24 h of culture. (h) Skin sensitivity responses on human arms after 1‐h contact with different materials. (i) Schematic illustration of the hemolysis test using rabbit blood. (j) Comparison of hemolysis ratios after 1‐h incubation.

The successful physicochemical properties of the sensor were verified through systematic characterization (Figure 2a–d). The Raman spectrum of the LIG exhibits three characteristic peaks of graphene: the D band located at 1324 cm^−1^, attributed to graphene edges and disordered structures; the G band located at 1586 cm^−1^, originating from the in‐plane vibration of sp^2^‐hybridized carbon atoms; and the 2D band located at 2706 cm^−1^, associated with interlayer stacking modes of carbon atoms (Figure S7). Energy‐dispersive X‐ray spectroscopy (EDS) analysis (Figure 2a,c) confirmed the effective attachment of I^−^ and Zn^2+^ ions on the electrode surface. The influence of LIG electroplating time on electrode performance is presented in Figure 2b, which reveals that the longer the time for plating, the higher the electrode conductivity. Further experiments on electroplating current density show that a moderate value of 30 mA/cm^2^ yields a dense and uniform zinc coating with optimal conductivity, while lower or higher current densities lead to insufficient deposition or dendritic growth, respectively, degrading the electrode performance (Figure S8). The iodine ion adsorption capability was evaluated using ultraviolet–visible (UV–vis) absorption spectroscopy (Figure 2d). Comparison between iodine aqueous solution and the iodine–CNT mixture showed that the addition of CNTs led to a clarified solution and a notable decrease in the characteristic absorption peaks of I^3^^−^ and I^5^^−^ species, directly demonstrating the efficient adsorption of iodine ions by CNT [40, 41, 42].

The breathability of the device is critical for wearing comfort, which was further evaluated in Figure 2e. A gravimetric method was employed to assess the moisture permeability of different materials. Results indicated that both the NF and the fabricated sensor device exhibited excellent breathability after 4 h of heating at 100°C, providing a fundamental basis for subsequent biocompatibility. A series of in vitro experiments were conducted to systematically evaluate the biocompatibility of the sensor and its core materials: Live/dead cell staining (Figure 2f–g) revealed that both the sensor group and the PVA–ZnCl_2_ hydrogel group maintained cell viability above 91% on the second day of culture, demonstrating excellent cytocompatibility. Skin sensitivity tests (Figure 2h) showed that no adverse skin reactions occurred after wearing the sensor for 1 h, whereas the non‐breathable PI film caused skin redness, highlighting the importance of breathability in minimizing irritation. Hemolysis assays using rabbit's blood (Figure 2i–j) indicated that the hemolysis rates of the sensor group and the PVA–ZnCl_2_ hydrogel group were both below the 5% safety threshold, confirming good hemocompatibility.

### 2.3 Simulation and Testing of Sensor Performance

**FIGURE 3:**
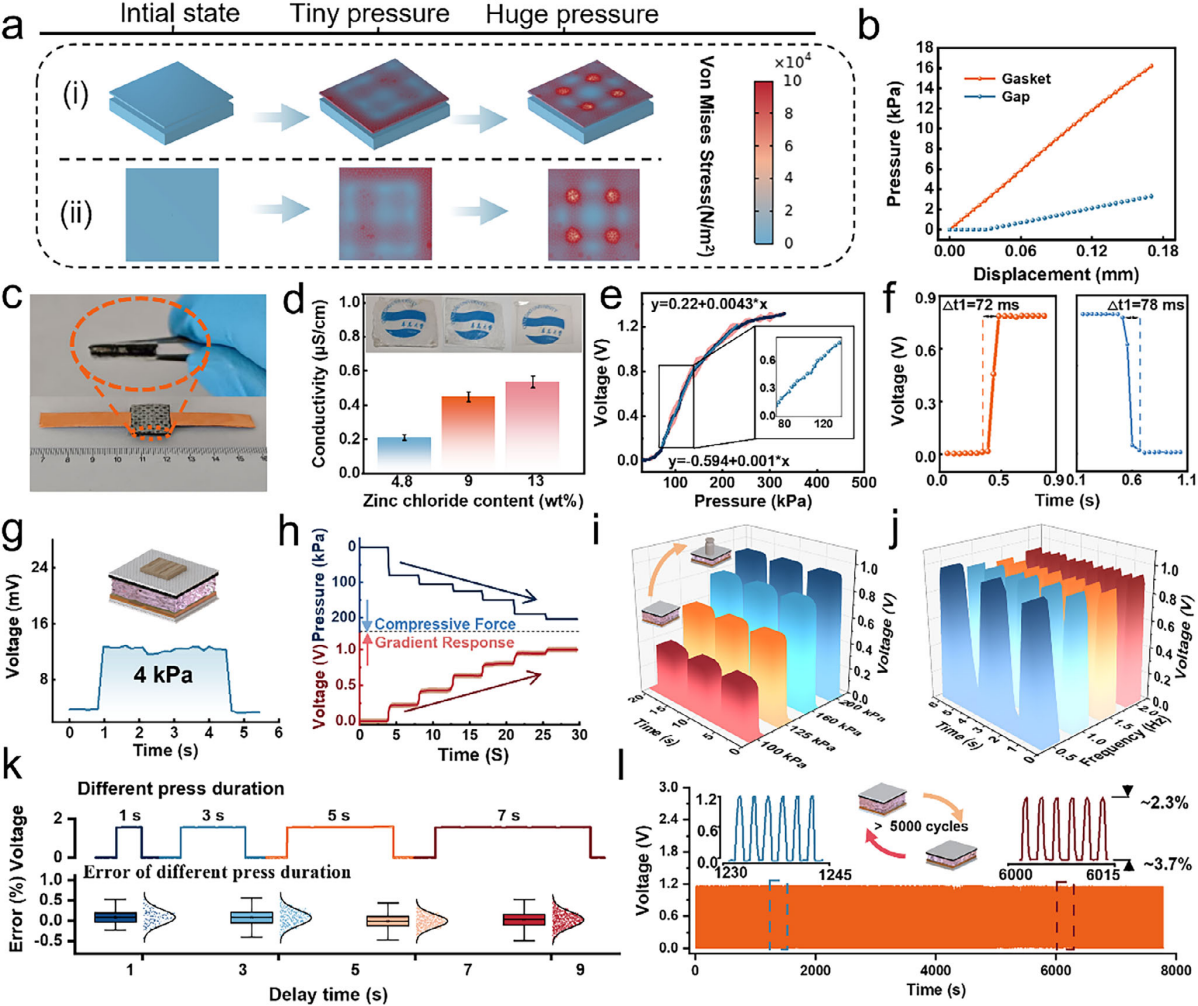
Sensor performance simulation and testing. (a) Finite element simulation of the mechanical behavior of the sensor and (b) corresponding simulation results. (c) Photograph of the fabricated sensor. (d) Conductivity of hydrogels with different ZnCl_2_ concentrations. (e) Sensitivity (voltage versus pressure) curve of the sensor. (f) Response and recovery time of the sensor. (g) Minimum detection limit of the sensor. (h) Gradient response of the sensor under different pressures. (i) Reproducibility of the sensor under various pressures. (j) Dynamic response of the sensor at different frequencies. (k) Continuous pressure response over different durations. (l) Long‐term cycling test of the sensor.

The mechanical behavior of the sensor was first analyzed via finite element simulation (Figure 3a). The simulation results suggest that under external pressure, the spacer first bears the load; with increasing pressure, the gap region is progressively stressed, leading to gradual contact between the electrode and the hydrogel electrolyte within that region (Figure 3b), effectively confirming that the spacer structure isolates the redox reaction in the initial state, ensuring a zero‐response voltage baseline under no‐load conditions (*p* = 0 Pa). The tensile and compressive properties of the sensor were also tested, revealing good mechanical behavior of the device (Figure S9). A photograph of the fabricated device is shown in Figure 3c, with an overall size of around 10 mm × 10 mm, where copper electrodes are used to connect the cathode and anode, respectively.

To optimize the electrolyte performance, the conductivity of PVA–ZnCl_2_ hydrogels with different ZnCl_2_ contents was measured (Figure 3d). The results show that the hydrogel conductivity increases significantly with higher ZnCl_2_ concentration; however, the hydrogel viscosity also increases (Figure S10), thus leading to a prolonged recovery time of the sensor. Based on this, a hydrogel with 13 wt.% ZnCl_2_ was ultimately selected as the electrolyte material to balance conductivity with other performance requirements. The sensitivity characteristics of the sensor over a broad pressure range of 0–300 kPa are shown in Figure 3e. Defining sensitivity as:

(1) S=ΔV/ΔP

where *V_0_* is the initial output voltage, Δ*V* represents the change in output voltage, and Δ*P* denotes the change in applied pressure. Two different linear responses are observed: in the low‐pressure range of 0–150 kPa, the sensitivity is 10 mV/kPa, while in the high‐pressure range of 150–256 kPa, it is 4.3 mV/kPa. This two‑segment behavior arises because below 150 kPa the electrode–electrolyte contact area increases linearly with pressure, whereas above 150 kPa the interface reaches full contact and further pressure only compresses the hydrogel, slowing the electrochemical response. This two‐segment behavior shows good repeatability across different devices; the small error bars from testing demonstrate that the pressure sensing performance is highly consistent among multiple samples. The device demonstrates excellent dynamic response performance, with both response time and recovery time below 100 ms (Figure 3f), showing its fast‐sensing capability. The minimum detection limit (Figure 3g) and gradient pressure response tests (Figure 3h) further confirm the sensor's ability to effectively resolve minor pressure variations and multiple pressure states.

The sensor exhibits outstanding operational stability. Under multiple pressure loading tests (Figure 3i), the output signals of the device are highly consistent, demonstrating high reliability. Furthermore, the sensor effectively responds to various low‐frequency dynamic stimuli (Figure 3j). Current responses under different pressures were also tested, showing similarly high stability (Figure S11). The average current sensitivity of the sensor is approximately 0.45 µA/kPa. Regarding the two output signals, the voltage signal offers excellent physicochemical stability and strong anti‑interference against environmental fluctuations; even after one week of ambient storage, its output decreased by only 0.26 V despite hydrogel dehydration. In contrast, the current signal avoids the saturation limitation of voltage signals under large pressures, because, according to Ohm's law, the continued decrease in resistance under pressure allows the current to respond continuously over a wide pressure range. The stability under static pressure was investigated by measuring responses under different pressing durations (Figure 3k). The violin plot shows that the device produces an almost constant output voltage under static stimulation, indicating excellent response consistency. Moreover, the device possesses remarkable durability (Figure 3l): after more than 5000 continuous cycles under 220 kPa pressure, no significant attenuation in the sensor output signal was observed, demonstrating a long operational lifetime and high reliability. When stored under ambient conditions for one week (Figure S12), daily tests showed a cumulative voltage decrease of 0.26 V, confirming good stability. Mass monitoring reveals that approximately 25% moisture loss during this period causes a decrease in ion mobility and an increase in contact resistance, which accounts for the observed voltage attenuation. Separate tests on environmental factors (Figure S13) revealed that humidity fluctuations between 35% and 80% relative humidity (RH) induced minimal output voltage variation (<0.02 V), indicating negligible humidity influence. In contrast, temperature variations between 15°C and 75°C led to a positive correlation with output voltage, with a slope of about 0.01 V per 10°C temperature increment. This temperature dependence may be attributed to the accelerated internal chemical reactions of the sensor at elevated temperatures.

### 2.4 Self‐Powered Mechanism and Electrochemical Performance of the Sensor

**FIGURE 4:**
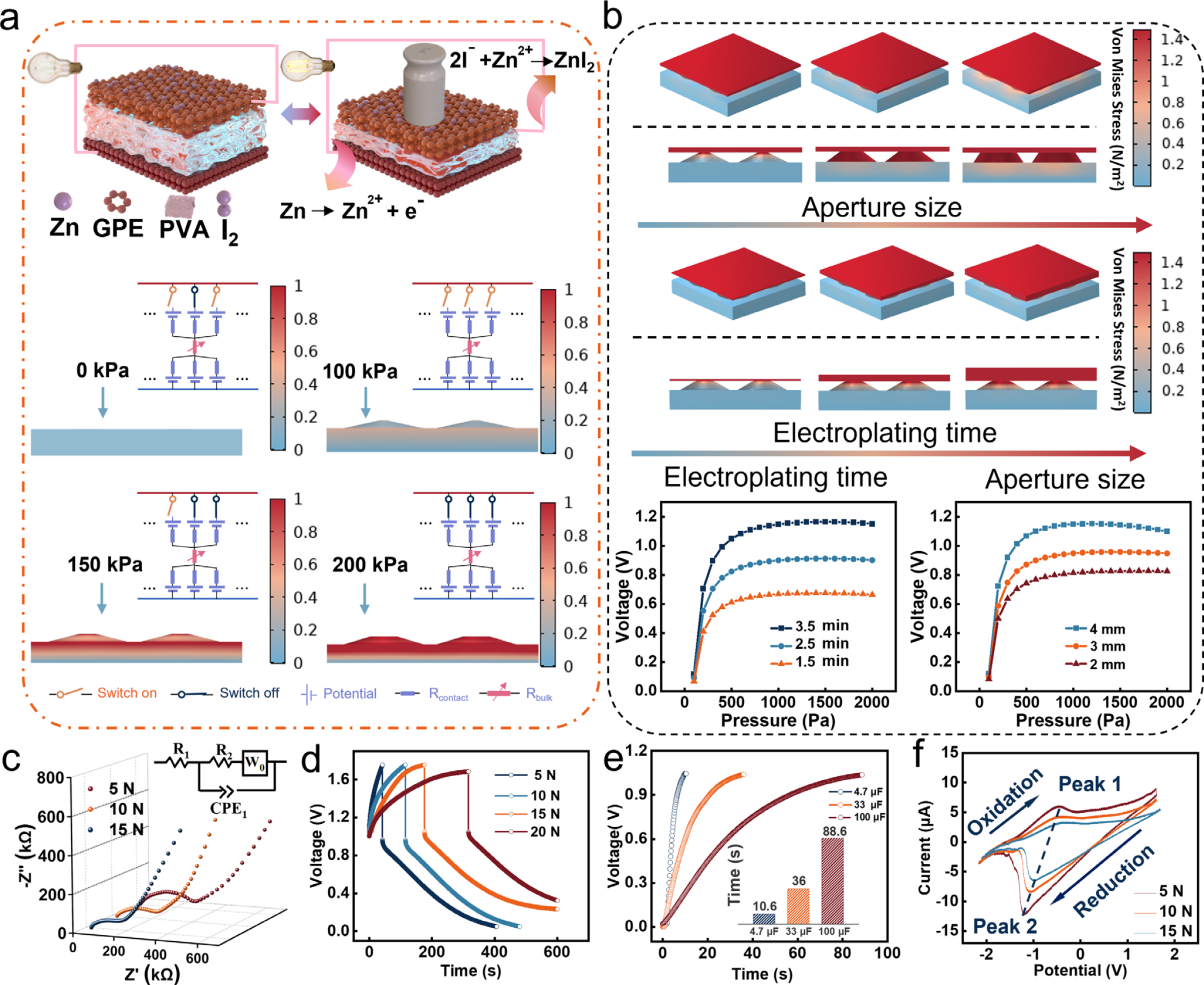
Self‐powered mechanism and electrochemical performance of the sensor. (a) Finite element simulation of the sensing mechanism. (b) Simulation results under different spacer openings and plating time. (c) EIS Nyquist plots under different pressures. (d) Variation of charging/discharging duration with applied pressure. (e) Charging time for different load capacitors. (f) Cyclic voltammetry (CV) curves measured at different pressure levels.

Finite element simulations show that external pressure deforms the PDMS spacer, bringing the PVA–ZnCl_2_ electrolyte into contact with the electrodes (Figure 4a). Initially, the electrolyte potential is uniform and zero (open circuit), preventing self‐discharge. As pressure increases, the contact area expands, homogenizing the potential in spacer void regions which increases ion flux and reduces interfacial/bulk impedance [37, 43, 44]. However, to understand why the output voltage rises from nearly zero to a saturated value, one must consider that the sensor is a galvanic cell: the measured terminal voltage *U* relates to the electromotive force *E*, internal resistance *r*, and current *I* by

(2) U=E−I·r

At zero pressure, poor contact makes *r* extremely large (near open circuit), so *U* ≈ 0 despite the existence of *E*. With increasing pressure, the growing contact area and opening of ion channels drastically reduce *r*, which increases *I* and lowers the *I*·*r* drop, thereby raising *U*. This process continues until the mechanical deformation reaches its limit: further reduction of *r* becomes negligible, and simultaneously *E* approaches its thermodynamic upper bound, causing *U* to saturate. Structural parameter simulations (Figure 4b) further confirm that enlarging the spacer aperture or increasing electrode thickness enhances the output voltage under the same pressure, as these modifications lower the internal resistance. To directly verify the “internal resistance dominated” mechanism, we added an external fixed resistor *R* (comparable to the initial *r*) across the sensor. The circuit satisfies

(3) $$U=\frac{E\times R}{R+r}$$

At low pressure, *r* ≫ *R* gives *U* **≈** 0; as pressure rises and *r* drops, *U* increases rapidly. When *r* becomes comparable to or smaller than *R*, further *r* reduction is limited and *E* saturates, so *U* no longer increases (Figure S14). This experiment confirms that the initial large internal resistance causes no voltage output, its decrease drives the voltage rise, and the saturation stems from the physical limits of both *r* and *E*.

Electrochemical impedance spectroscopy (EIS) measurements with varied pressures (Figure 4c) also give evidence for the above proposed mechanism. The characteristic semicircle in the mid‐frequency region primarily corresponds to the charge transfer process at the electrolyte/electrode interface (electrochemical reaction kinetics), and its radius decreases markedly with increasing pressure. Simultaneously, the tail in the low‐frequency region reflects ion diffusion behavior within the electrode active material. The EIS results consistently demonstrate that the total impedance of the device decreases systematically with increasing applied pressure, highly consistent with the simulated pressure‐impedance relationship.

The dynamics in charging/discharging processes exhibit a strong pressure dependence (Figure 4d). As the applied pressure increases, the output voltage rises correspondingly, while the time of one charge‐ discharge cycle is extended as well. The device also shows excellent stability in energy storage output, Galvanostatic charge/discharge profiles at different current densities and two consecutive capacitor charging cycles (Figure S15) confirm its reliable performance. When charging capacitors of varying capacitances (Figure 4e), the output voltage remains stable with no decline during charging. These results verify that the proposed device is suitable for use both as a micro‐power source and as a pressure sensor. The load‐driving capability was also measured (Figure S16), exhibiting favorable response characteristics under various load conditions. The CV tests (Figure 4f) clearly display reversible redox peaks, which indicates that the desired reaction occurs on the electrodes’ surface as clear proof of the chemistry involved during the whole detection procedure.

Subsequently, sensors were connected in series to test the total output voltage (Figure S17). The voltage from two serially connected devices reached approximately 2.5 V. A sensor array was fabricated and can be used directly as a power supply to drive an LED lamp (Figure S18 and Video S1), whose on/off state was modulated by the magnitude of the applied pressure. For more flexible control, a charging capacitor was incorporated into the system. The array was first pressed to charge the capacitor (switch is open, LED remains off); subsequently, a single‐pole double‐throw (SPDT) switch was closed to allow the capacitor to discharge through the LED and illuminate it (Video S2). The capacitor charged by such an array was also able to power up a small electronic meter (Figure S19).

### 2.5 Applications

#### 2.5.1 Sensor Application in User Identification System

**FIGURE 5:**
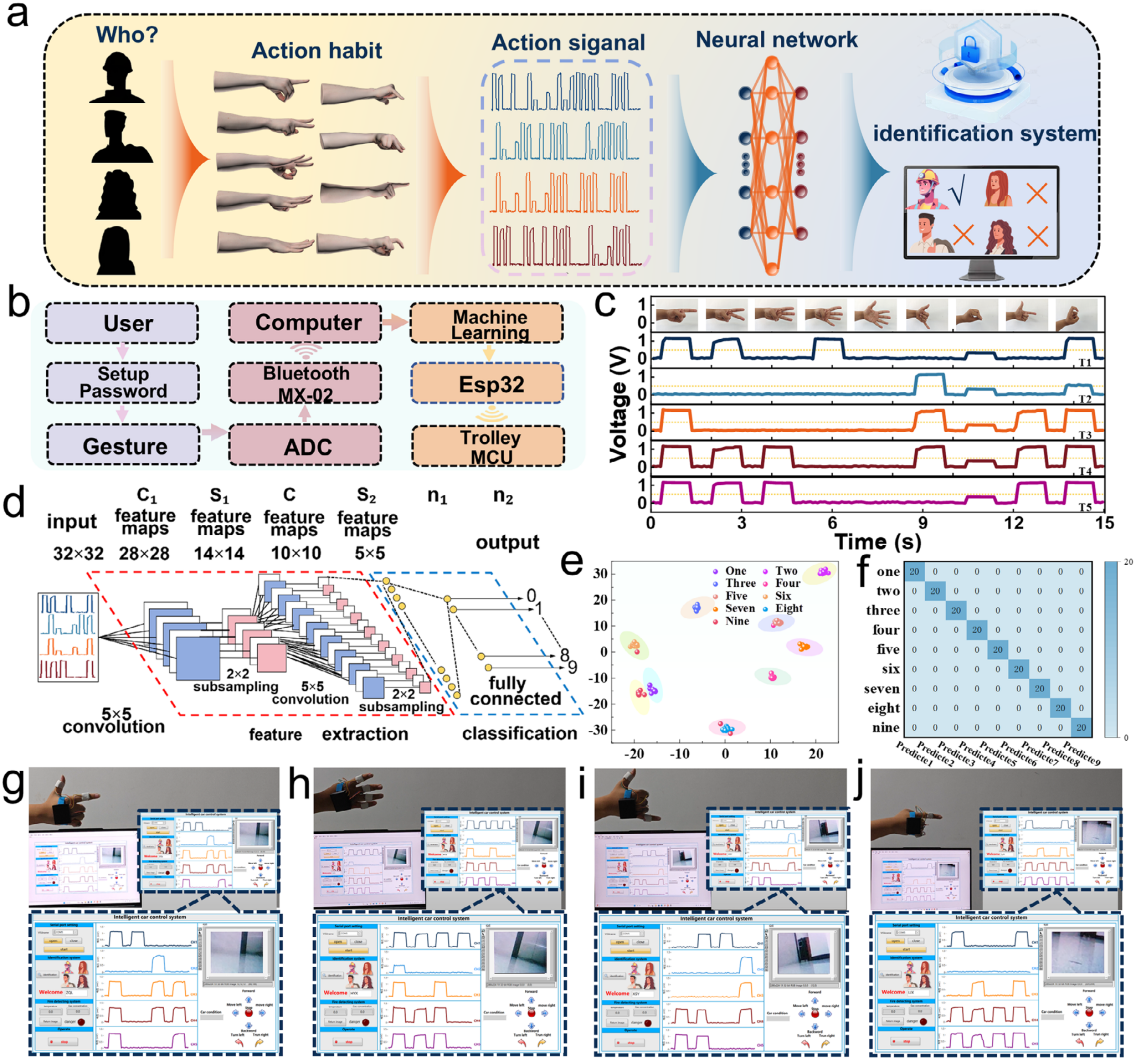
Sensor application in user identification system. (a) Schematic diagram of the system recognition process. (b) Workflow of the user identification system. (c) Signal curves of different collected gesture motions. (d) Architecture of the employed Convolutional Neural Network (CNN) model. (e) PCA classification results. (f) Confusion matrix of the machine learning model. (g–j) User identification system based on different gesture characteristics.

The growing demand for biosecurity has motivated the development of a biometric authentication system based on gesture recognition, which ensures exclusive vehicle access for authorized users and prevents unauthorized activation. As illustrated in Figure 5a, the system first constructs a personalized gesture database: multiple subjects wearing the sensor glove were asked to perform distinct dynamic gesture sequences, with only one authorized user performing the preset gesture sequence (Video S3). He collected multi‐dimensional sensor signals were preprocessed and fed into a neural network for feature extraction and pattern recognition. Identity verification is achieved by comparing the real‐time gesture waveform with the preregistered template of the authorized user.

The system workflow is depicted in Figure 5b: once the owner has defined his own gesture password, any user attempting to start the vehicle must reproduce a specific gesture sequence. The dynamic pressure signals generated by finger movements are acquired by the sensors and transmitted to a host computer, where a trained neural network model performs feature analysis and decision‐making by analyzing the dynamic pressure patterns. The host computer sends an unlock command through the transmission control protocol / internet protocol (TCP/IP) only if the recognition results match with the preset gesture of the authorized user, thus, enabling the integrated safety control.

In order to ensure model robustness, a high‐quality gesture database was established. As shown in Figure 5c, sufficient samples of each gesture were collected to ensure data consistency. Different waveforms appear in different gestures either in the time domain or the frequency domain. During the machine learning modeling phase using a CNN (Figure 5d), the dataset was randomly split into training (70%) and test (30%) sets, principal component analysis (PCA) was employed to reduce the dimensionality of high‐dimensional features (Figure 5e) [45, 46]. The PCA visualization clearly showed well‐separated cluster of features for each gesture, verifying that each gait is unique. The model performance was rigorously validated by a confusion matrix (Figure 5f), which demonstrated 100.0% recognition accuracy on the test set, indicating excellent generalization capability.

Physical verification of the system (Figure 5g–j) was conducted through a host computer interface that displays real‐time gesture waveforms and recognition results. When a user performs the gesture sequence, the system simultaneously visualizes the sensor data stream. If the continuous gesture sequence matches the preset password, the interface displays the corresponding user's name, providing intuitive feedback for completed identity authentication; if the sequence does not match, the interface shows “error” and the vehicle remains locked (Figure S20). By integrating biometric recognition with physical control, this system offers a highly secure and low false‐acceptance solution for proactive protection of mobile vehicles.

#### 2.5.2 Sensor Application in Intelligent Fire Trolley Control System

Current mainstream control methods for remote detection vehicles primarily rely on handheld remote controls or smartphone applications. Although these technologies are relatively mature, their operational convenience remains limited in practical fire reconnaissance scenarios. To simplify the control process and improve operational efficiency, gesture recognition‐based interaction technology is emerging as a research focus. Wearable gesture recognition devices can be used for this purpose (e.g., smart glove), a firefighter is able to command the detection vehicle via making simple hand gestures This approach not only keeps the operator's hands free but also significantly enhances operational flexibility and system reliability in complex fire environments. It provides an alternative means of mobile intuitive control for fire reconnaissance applications where the environment is potentially dangerous with heavy smoke, high temperatures, or many barriers.

**FIGURE 6:**
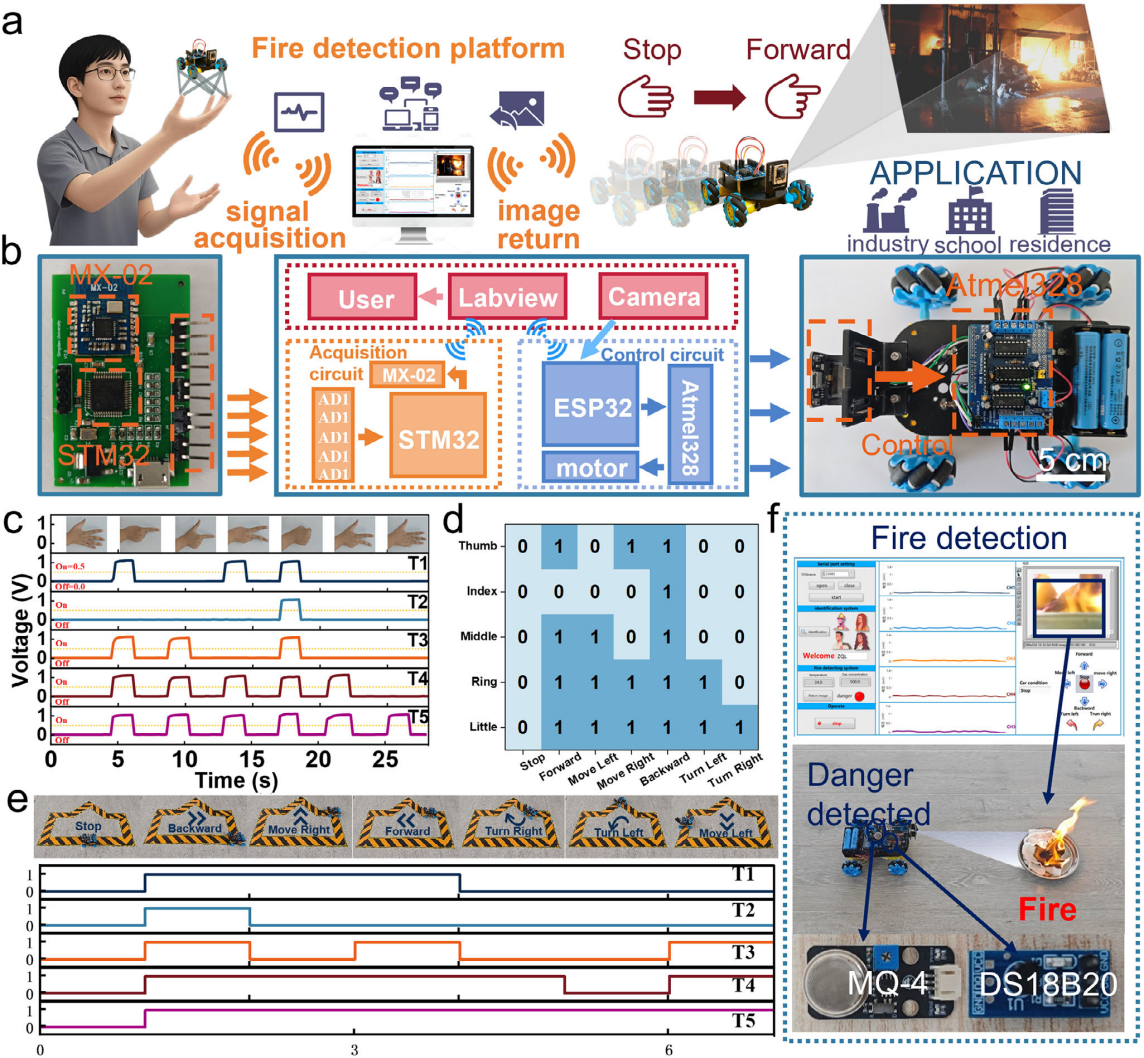
Sensor application in an intelligent fire trolley control system. (a) Schematic of the system based on the smart glove. (b) Hardware communication and data processing framework of the system. (c) Raw sensor signal waveforms corresponding to different gestures. (d) Gesture‐to‐command mapping based on the threshold method. (e) Physical validation scenario demonstrating gesture‐controlled vehicle operation. (f) Schematic of the multi‐sensor fire detection system.

As shown in Figure 6a, this study developed a vehicle control system based on gesture recognition. The operator wears a smart glove integrated with multi‐channel sensors, generating control commands by performing specific gestures. Analog signals collected by the glove are synchronously processed through five channels on an embedded acquisition board (Figure 6b), digitized by an Analog‐to‐Digital Converter (ADC) module, and transmitted to a LabVIEW host computer via an MX–02 Bluetooth module. The host computer employs a threshold algorithm to parse gesture features in real time (Figure 6c–d): a signal voltage >0.5 V is recorded as logic “1”, otherwise “0”, thereby generating a control command mapping table (e.g., Channel 1>0.5 V triggers the forward command). Commands are sent wirelessly to the vehicle's actuation system, enabling real‐time translation of gestures into motion (Figure 6e; Video S4). Environmental awareness is established through the communication between the smart car and the operator; the onboard camera transmits images in real time from the smart car's environment into the laptop screen, allowing the operator to remotely observe complex scenarios (e.g., schools, factories, apartments, and other crowded areas). Simultaneously, an integrated fire detection subsystem (Figure 6f) equipped with smoke and temperature sensors automatically triggers a red alarm indicator on the host interface when abnormal thresholds are detected, while the visual module displays the fire location. By integrating gesture control, environmental monitoring, and visual feedback, this system effectively enhances the productivity and rescue performance in complex environments.

## 3 Conclusion

In conclusion, we successfully developed a self‐powered potentiometric pressure sensor based on Zn–I_2_ electrochemistry, capable of sensing the dynamic and static pressure. The sensor has a flexible and breathable structure, with an electrolyte layer and functionalized electrodes as the core components. External pressure modulates the electrode–electrolyte contact interface, altering the electrochemical reaction state and generating a stable open‐circuit voltage signal. Owing to its rational structural design, the sensor demonstrates outstanding overall performance, including an ultrafast response/recovery speed, excellent long‐term stability, and remarkable environmental adaptability. Besides, the breathability and biocompatibility of the device were experimentally verified, ensuring comfort and safety for wearable applications. Based on this high‐performance sensor, we further constructed an intelligent glove system integrated with multiple sensing units. By combining a dynamic threshold analysis algorithm and wireless communication technology, the system achieves high‐accuracy gesture recognition. It has been successfully applied to the remote wireless control of a fire‐detection smart car equipped with multi‐functional environmental monitoring modules (e.g., temperature/gas sensors, camera), to form a closed‐loop human‐machine interaction framework that integrates “gesture command‐environmental monitoring”. This integrated system not only enhances operational flexibility and efficiency in complex emergency scenarios but also enables real‐time perception and feedback of multi‐modal information under dangerous environments, showing its broad application prospects in smart wearable fields.

## 4 Experimental Section

### 4.1 Fabrication of LIG Electrodes

A PI film with a thickness of 125 µm was used as the original substrate. The local carbonization was performed on the PI film surface using a carbon dioxide laser engraving machine (KB–K3020, Liaocheng Keba Laser Equipment Co., Ltd., China) with a power of 14 W and a scanning speed of 250 mm/s to accurately engrave a square LIG electrode pattern with a length of 1 mm. By taking advantage of the intrinsic surface adhesiveness (pressure‐sensitive acrylic adhesive), the LIG electrode was transferred onto this flexible and breathable medical‐grade NF substrate. Due to the microscale adhesion generated by the fibrous structure of the NF, the LIG layer effectively peeled off the PI film and tightly bonded with the surface of the NF. Afterward, peeling off the PI film resulted in a NF‐based electrode platform, loaded with a complete square LIG conducting pattern.

### 4.2 Preparation of Iodine Electrode

Iodine (I_2_, 0.5 g, Shanghai Aladdin Biochemical Technology Co., Ltd.), multi‐walled carbon nanotubes (MWCNTs, 1.0 g, Shanghai Aladdin Biochemical Technology Co., Ltd.), acetylene black (0.1 g, Songhu Shenjian Technology Co., Ltd.), and glycerol (C_3_H_8_O_3_, 0.3 g, Shanghai Macklin Biochemical Technology Co., Ltd.) were weighed respectively. The mixture was placed on a magnetic stirrer (CMVC–M003H) at 100°C and stirred continuously for 1 h to ensure thorough mixing and promote the adsorption of iodine onto the carbon material surface. The mixture was then cooled down and stood at room temperature for 2 h, forming a homogeneous and stable black ink paste. A pre–fabricated screen–printing stencil with a square electrode pattern (side length of 1 mm) was carefully positioned on the LIG electrode area previously transferred onto the NF. Subsequently, the prepared iodine composite ink was uniformly coated onto the stencil, then printing was conducted by using a scraper with uniform force, allowing for the ink to deposit through the mesh on top of the target LIG region. After that, the stencil was carefully removed, obtaining a cathode loaded with the I_2_/CNT/acetylene black composite.

### 4.3 Preparation of Zinc Electrode

A 0.1 m aqueous zinc chloride (ZnCl_2_) solution was used as the electroplating solution. The NF substrate with the transferred LIG electrode served as the working electrode (cathode), which was dipped into the electroplating solution. A high‐purity zinc sheet was used as the counter electrode (anode). Alligator clips were used to connect the working and counter electrodes to the power supply. The electrochemical reaction under room temperature condition is operated in the constant current mode at a current density of 30 mA/cm^2^ (calculated based on the area of the target electrode), and the deposition time was controlled to be 3.5 min. During the electrodeposition process, Zn^2+^ ions dissolved in the solution were reduced on the LIG substrate surface, forming a homogeneous and dense metallic zinc film (Zn/LIG).

### 4.4 Fabrication of the Hydrogel Electrolyte Layer

The solid–state electrolyte layer utilized a PVA/ZnCl_2_ hydrogel, which was fabricated in following steps: First, 1.0 g of PVA powder (Sinopharm Chemical Reagent Co., Ltd.), 1.5 g of anhydrous ZnCl_2_ (analytical grade, Sinopharm Chemical Reagent Co., Ltd.), and 9.0 g of deionized water were weighed and placed in a reaction container. Secondly, the mixture was placed on a magnetic stirrer and stirred continuously for 2 h to ensure full swelling and gradual dissolution of PVA particles, while allowing complete dissociation of ZnCl_2_. Thirdly, to thoroughly remove bubbles generated during stirring and ensure complete dissolution of PVA, the resulting viscous solution was transferred to a 90°C electric blast drying oven (model: DHG–9070A) and heated for 1 h, ultimately yielding a homogeneous, transparent, bubble‐free PVA/ZnCl_2_ mixed solution. Fourthly, the precursor solution was carefully poured into a polystyrene culture dish (diameter approx. 90 mm), controlling the pouring process to achieve a liquid layer of uniform thickness. Fifthly, the cast culture dish was then placed at room temperature and atmospheric pressure to stand for 36 h. During this period, water slowly evaporated, and the PVA molecular chains formed a 3D network structure through physical entanglement and hydrogen bonding, while Zn^2+^ and Cl^−^ ions were effectively confined within this hydrophilic polymer network, finally solidifying into a semi‐transparent solid hydrogel film with certain mechanical strength and elasticity. Finally, after the hydrogel was fully formed, clean tweezers were used to peel off and remove the entire hydrogel film from the edge of the culture dish. According to the experimental device requirements, a blade was used to precisely cut the hydrogel into specific‐sized squares (typical working size 1 cm × 1 cm), with a measured thickness of approximately 0.64 mm.

### 4.5 Sensor Characterization and Testing

An electric tension and compression testing machine (ZQ–990B) and a digital multimeter (KEITHLEY–DMM6500) have been used to measure the forces and voltages, respectively. An electrochemical workstation (CHI–CHI660E) was employed to study electrochemical performance. An SEM (TESCAN MIRA) was applied to characterize the microstructure and morphology of the zinc–iodine square electrode and hydrogel. A HORIBA LabRAM HR Evolution spectrometer was used to record the Raman spectra of the LIG.

### 4.6 Cytotoxicity Assessment

Mouse fibroblast cells (L929) were used for cytotoxicity evaluation. L929 cells were seeded in a 24‐well plate at a density of 4 × 104 cells/well and cultured for 24 h. The hydrogel and the integrated device were immersed in Dulbecco's Modified Eagle Medium (DMEM) medium at a concentration of 10 mg/mL and incubated for 24 h. Subsequently, the conditioned medium from the material culture was added to the 24‐well plate and the L929 cells were cultured for 1 day and 2 days. Live/dead staining was performed on the L929 cells cultured for 1 day and 2 days. For staining, 5 µL of propidium iodide and 5 µL of Calcein AM were added to 10 mL of phosphate buffered saline (PBS) working solution; 100 µL of this working solution was added to each well. After staining for 30 min, the wells were washed 2–3 times with PBS to remove the working solution, and the staining situation was observed using a fluorescence microscope. All experiments were independently repeated three times.

### 4.7 Hemolysis Assay

Rabbit fresh anticoagulated blood was washed three times with PBS (5000 rpm, 5 min each), the red blood cells were resuspended in 10 mL of PBS to prepare a red blood cell suspension. The experimental groups were set up as follows: positive (+) control (200 µL suspension + 800 µL deionized water), negative (−) control (200 µL suspension + 800 µL PBS), experimental group 1 (200 µL suspension + 800 µL PBS + 50 mg hydrogel), and experimental group 2 (200 µL suspension + 800 µL PBS + 50 mg integrated device). All groups were incubated at 37°C for 1 h, then centrifuged at 10 000 rpm for 5 min. 100 µL of the supernatant from each group was transferred to a 96‐well plate, then measure its absorbance at 540 nm using a microplate reader. All experiments were independently repeated three times. The hemolysis rate (%) was calculated according to the formula:

(4) $$Hemolysis\;rate\;\left(\%\right)=\frac{Ab-Ab\left(-\right)}{Ab\left(+\right)-Ab\left(-\right)}\times 100\%$$

where, *Ab* denotes the absorbance of the red blood cell suspension treated with the hydrogel or integrated device, *Ab*(+) and *Ab*(−) refer to the absorbance values of red blood cell suspensions treated with deionized water (positive control) and sterile PBS (negative control), respectively.

### 4.8 Intelligent Firefighting Vehicle Control System

In order to realize portable detection in the scene of the fire, an intelligent firefighting vehicle control system was developed. This system adopted a distributed architecture, with the core perception unit consisting of multi‐sensor devices and their acquisition board. The acquisition board was equipped with an STM32 microprocessor, responsible for real‐time acquisition of five‐channel data. The acquired data was wirelessly transmitted to a remote monitoring terminal via an onboard MX–02 Bluetooth module. The terminal, running host computer software developed on the LabVIEW platform, received the data and communicates instructions via the TCP/IP protocol with an ESP32–CAM module deployed on the firefighting vehicle. The firefighting vehicle platform integrated an MQ–4 smoke sensor and a DS18B20 digital temperature sensor to monitor the on‐site smoke concentration and ambient temperature. In addition, the ESP32–CAM module integrated an OV2640 vision sensor, constituting a visual perception module that transmits live image data back to the host computer. With the combination of vision information, smoke density, and thermal information, such a control system realized multimodal collaborative detection for the scene of the fire, significantly enhancing the accuracy and comprehensiveness of on‐site fire situation awareness.

## Conflicts of Interest

The authors declare no conflict of interest.

## Supporting information




**Supporting File 1**: advs75660‐sup‐0001‐SuppMat.docx.

**Supporting File 2**: advs75660‐sup‐0002‐VideoS1‐S4.zip.

## Data Availability

The data that support the findings of this study are available from the corresponding author upon reasonable request.

## References

[1] X. Li , T. Li , Y. Liu , et al., “Non‐Loss Engraved Circuit Patterning Method of Semi‐Liquid Metal for Precision Recyclable Multi‐Substrate Circuits,” Nature Communications 16 (2025): 11690, 10.1038/s41467-025-66815-4. PMC12748806 41309596

[2] L. Zhang , L. Chen , S. Wang , et al., “Cellulose Nanofiber‐Mediated Manifold Dynamic Synergy Enabling Adhesive and Photo‐Detachable Hydrogel for Self‐Powered E‐Skin,” Nature Communications 15 (2024): 3859, 10.1038/s41467-024-47986-y. PMC11078967 38719821

[3] Z. Yang , Q. Wang , H. Yu , et al., “Self‐Powered Biomimetic Pressure Sensor Based on Mn–Ag Electrochemical Reaction for Monitoring Rehabilitation Training of Athletes,” Advanced Science 11 (2024): 2401515, 10.1002/advs.202401515. 38654624 PMC11220713

[4] X. Liu , Y. Li , Y. Li , et al., “Bionic Fingerprint Tactile Sensor With Deep Learning‐Decoupled Multimodal Perception for Simultaneous Pressure‐Friction Mapping,” Advanced Functional Materials 35 (2025): 06158, 10.1002/adfm.202506158.

[5] L. Wu , J. Huang , Y. Chen , et al., “Breathable and Highly Sensitive Self‐Powered Pressure Sensors for Wearable Electronics and Human‐Machine Interaction,” Composites Science and Technology 262 (2025): 111078, 10.1016/j.compscitech.2025.111078.

[6] S. Guo , S. Patel , J. Wang , et al., “Self‐Powered Green Energy–Harvesting and Sensing Interfaces Based on Hygroscopic Gel and Water‐Locking Effects,” Science Advances 11 (2025): adw5991, 10.1126/sciadv.adw5991. PMC12219473 40601730

[7] Q. Wang , H. Guan , C. Wang , et al., “A Wireless, Self‐Powered Smart Insole for Gait Monitoring and Recognition Via Nonlinear Synergistic Pressure Sensing,” Science Advances 11 (2025): adu1598, 10.1126/sciadv.adu1598. PMC12002114 40238890

[8] P. Wang , G. Wang , G. Sun , et al., “A Flexible‐Integrated Multimodal Hydrogel‐Based Sensing Patch,” Nano‐Micro Letters 17 (2025): 156, 10.1007/s40820-025-01656-w. 39982550 PMC11845634

[9] S.‐Z. Liu , W.‐T. Guo , X.‐H. Zhao , X.‐G. Tang , and Q.‐J. Sun , “Self‐Powered Sensing for Health Monitoring and Robotics,” Soft Science 5 (2025): 14, 10.20517/ss.2024.65.

[10] Z. Lin , Y. Wang , S. Nie , et al., “Island‐Bridge Microstructured Triboelectric Pressure Sensor for Effective Dynamic Epidermal Pulse Monitoring,” Soft Science 5 (2025): 47, 10.20517/ss.2025.53.

[11] S. Meng , H. Yi , K. Ge , et al., “Additively Manufactured Flexible Electronics Filled With Ionic Liquid for Cryogenic Pressure Sensing,” Advanced Devices & Instrumentation 5 (2024): 0052, 10.34133/adi.0052.

[12] X. Wu , Y. Liang , L. Lu , et al., “Fabric‐Based Flexible Pressure Sensor Arrays With Ultra‐Wide Pressure Range for Lower Limb Motion Capture System,” Research 8 (2025): 0835, 10.34133/research.0835. 40832479 PMC12358749

[13] H. Sun , L. Li , L.‐Q. Tao , et al., “An Intelligent Multifunction Graphene Skin Patch for Ear Health Monitoring and Acoustic Interaction,” Nano Energy 137 (2025): 110790, 10.1016/j.nanoen.2025.110790.

[14] S.‐Y. Xia , L.‐Y. Guo , Y. Long , W. Chen , and J. Li , “Integrated Sensing–Memory–Computing Artificial Tactile System Based on Force Sensors and Memristors,” Applied Physics Letters 122 (2023): 183504, 10.1063/5.0149271.

[15] L. Yang , X. Chen , A. Dutta , et al., “Thermoelectric Porous Laser‐Induced Graphene‐Based Strain‐Temperature Decoupling and Self‐Powered Sensing,” Nature Communications 16 (2025): 792, 10.1038/s41467-024-55790-x. PMC11742402 39824812

[16] H. Ren , W. Li , H. Li , et al., “Jellyfish‐Inspired High‐Sensitivity Pressure‐Temperature Sensor,” Advanced Functional Materials 35 (2025): 2417715, 10.1002/adfm.202417715.

[17] L. Wu , X. Li , J. Choi , et al., “Beetle‐Inspired Gradient Slant Structures for Capacitive Pressure Sensor With a Broad Linear Response Range,” Advanced Functional Materials 34 (2024): 2312370, 10.1002/adfm.202312370.

[18] P. Wang , C. Zhang , B. Li , et al., “Hypersensitive Pressure Sensors Inspired by Scorpion Mechanosensory Mechanisms for Near‐Body Flow Detection in Intelligent Robots,” Science Advances 11 (2025): ady5008, 10.1126/sciadv.ady5008. PMC12366690 40834079

[19] H. Wang , D. Ruan , L. Gan , et al., “A Capacitive Pressure Sensor With Adjustable Working Range for Deep Sea,” IEEE Sensors Journal 24 (2024): 37005–37014, 10.1109/JSEN.2024.3464608.

[20] H. Yu , Z. Hu , J. He , et al., “Flexible Temperature‐Pressure Dual Sensor Based on 3D Spiral Thermoelectric Bi_2_Te_3_ Films,” Nature Communications 15 (2024): 2521, 10.1038/s41467-024-46836-1. PMC10958038 38514626

[21] R. B. Mishra , N. E. Atab , A. M. Hussain , and M. M. Hussain , “Recent Progress on Flexible Capacitive Pressure Sensors: From Design and Materials to Applications,” Advanced Materials 6 (2021): 2001023, 10.1002/admt.202001023.

[22] G. Xu , H. Wang , G. Zhao , et al., “Self‐Powered Electrotactile Textile Haptic Glove for Enhanced Human‐Machine Interface,” Science Advances 11 (2025): adt0318, 10.1126/sciadv.adt0318. PMC11927614 40117358

[23] M. Guo , Y. Xia , J. Liu , Y. Zhang , M. Li , and X. Wang , “Wearable Pressure Sensor Based on Triboelectric Nanogenerator for Information Encoding, Gesture Recognition, and Wireless Real‐Time Robot Control,” Advanced Functional Materials 35 (2025): 2419209, 10.1002/adfm.202419209.

[24] C. Xue , Y. Zhao , Y. Liao , and H. Zhang , “Bioinspired Super‐Robust Conductive Hydrogels for Machine Learning‐Assisted Tactile Perception System,” Advanced Materials 37 (2025): 2416275, 10.1002/adma.202416275. 39901430

[25] W. Liu , Z. Du , Z. Duan , et al., “Eye‐Wearable Ti_3_C_2_T_x_ MXene‐Based Micro‐Supercapacitor as a Power Unit for Intraocular Pressure Applications,” Journal of Materials Chemistry A 12 (2024): 16457–16465, 10.1039/D4TA02127D.

[26] T. Huang , B. Gao , M. Li , et al., “Cathode‐Free Aqueous Micro‐battery for an All‐in‐One Wearable System With Ultralong Stability,” Advanced Energy Materials 15 (2025): 2402871, 10.1002/aenm.202402871.

[27] J. Chen , Y. Zhou , T. Song , et al., “Ultralow Voltage Operation and Microwatt Power Consumption of MXene‐Based Pressure Sensors With Excellent Sensing Performance,” Journal of Materials Chemistry A 13 (2025): 6539–6548, 10.1039/D4TA08386E.

[28] A. Wang , Z. Gao , S. Wu , et al., “Superelastic and Ultra‐Soft MXene/CNF Aerogel@PDMS‐Based Dual‐Modal Pressure Sensor for Complex Stimuli Monitoring,” Advanced Science 12 (2025): 2502797, 10.1002/advs.202502797. 40192090 PMC12244993

[29] Z. Huang , Z. Duan , Q. Huang , Z. Yuan , Y. Jiang , and H. Tai , “A Facilely Fabricated Electrochemical Self‐Powered Pressure Sensor for Multifunctional Applications,” Journal of Materials Chemistry C 12 (2024): 18320–18326, 10.1039/D4TC03434A.

[30] Q. Zhang , D. Lei , J. Shi , et al., “Pressure‐Regulated Nanoconfined Channels for Highly Effective Mechanical–Electrical Conversion in Proton Battery‐Type Self‐Powered Pressure Sensor,” Advanced Materials 35 (2023): 2308795, 10.1002/adma.202308795. 37967569

[31] S. Kim , W. Cho , J. Hwang , and J. Kim , “Self‐Powered Pressure Sensor for Detecting Static and Dynamic Stimuli Through Electrochemical Reactions,” Nano Energy 107 (2023): 108109, 10.1016/j.nanoen.2022.108109.

[32] D. Lei , Q. Zhang , N. Liu , et al., “An Ion Channel‐Induced Self‐Powered Flexible Pressure Sensor Based on Potentiometric Transduction Mechanism,” Advanced Functional Materials 32 (2022): 2108856, 10.1002/adfm.202108856.

[33] X. Wen , Z. Sun , X. Xie , et al., “High‐Performance Fully Stretchable Moist‐Electric Generator,” Advanced Functional Materials 34 (2024): 2311128, 10.1002/adfm.202311128.

[34] Q. Zhang , D. Lei , N. Liu , et al., “A Zinc‐Ion Battery‐Type Self‐Powered Pressure Sensor With Long Service Life,” Advanced Materials 34 (2022): 2205369, 10.1002/adma.202205369. 35986663

[35] X. Wu , J. Zhu , J. W. Evans , C. Lu , and A. C. Arias , “A Potentiometric Electronic Skin for Thermosensation and Mechanosensation,” Advanced Functional Materials 31 (2021): 2010824, 10.1002/adfm.202010824.

[36] X. Wu , J. Zhu , J. W. Evans , and A. C. Arias , “A Single‐Mode, Self‐Adapting, and Self‐Powered Mechanoreceptor Based on a Potentiometric–Triboelectric Hybridized Sensing Mechanism for Resolving Complex Stimuli,” Advanced Materials 32 (2020): 2005970, 10.1002/adma.202005970. 33179325

[37] X. Dai , J. Zou , X. Liu , et al., “Mechanical‐Electrochemical Conversion for Self‐Powered Sensing and Alterable Power Supply,” Materials Science and Engineering: R: Reports 163 (2025): 100892, 10.1016/j.mser.2024.100892.

[38] A. Cheng , X. Li , D. Li , et al., “An Intelligent Hybrid‐Fabric Wristband System Enabled by Thermal Encapsulation for Ergonomic Human‐Machine Interaction,” Nature Communications 16 (2025): 591, 10.1038/s41467-024-55649-1. PMC11724971 39799116

[39] W. Qin , Y. Xue , G. Li , et al., “Highly‐Sensitive Wearable Pressure Sensor Based on AgNWs/MXene/Non‐Woven Fabric,” Organic Electronics 125 (2024): 106958, 10.1016/j.orgel.2023.106958.

[40] L. Zhu , X. Guan , Y. Fu , et al., “Integrated Trap‐Adsorption‐Catalysis Nanoreactor for Shuttle‐Free Aqueous Zinc‐Iodide Batteries,” Advanced Functional Materials 34 (2024): 2409099, 10.1002/adfm.202409099.

[41] F. Wang , R. Ma , Z. Chen , et al., “Click Chemistry‐Inspired Fixation Catalysis for Long‐Life Zinc–Iodine Batteries,” Advanced Materials 38 (2026): 11980, 10.1002/adma.202511980. 40964936

[42] H. Wu , S. Zhang , J. Vongsvivut , Y. Jiang , J. Hao , and S. Qiao , “Quasi‐Solid Cathode Additive Enables Highly Reversible Four‐Electron I^−^/I^0^/I^+^Conversion in Aqueous Zn‐I_2_ Batteries,” Advanced Materials 38 (2026): 11680, 10.1002/adma.202511680. PMC12801367 41030210

[43] T. Zhang , F. Manshaii , C. R. Bowen , et al., “A Flexible Pressure Sensor Array for Self‐Powered Identity Authentication During Typing,” Science Advances 11 (2025): ads2297, 10.1126/sciadv.ads2297. PMC11900873 40073146

[44] X. Zhao , Y. Li , K. Chen , et al., “Completely Flexible Self‐Powered Pressure Sensor Based on Electrospinning and Electrochemical Reaction for Dynamic/Static Stimuli Detecting,” ACS Sensors 10 (2025): 1011–1022, 10.1021/acssensors.4c02824. 39964735

[45] Y. Lei , D. Zhang , Q. Wang , et al., “Detection of Carcinoembryonic Antigen Specificity Using Microwave Biosensor With Machine Learning,” Biosensors and Bioelectronics 269 (2025): 116908, 10.1016/j.bios.2024.116908. 39549313

[46] J. Tao , J. Huang , J. Tong , et al., “Deep Learning‐Enabled Self‐Powered Stretchable Triboelectric Sensor Array for Intelligent Posture Monitoring and Regulation,” Advanced Functional Materials 36 (2025): 14646, 10.1002/adfm.202514646.

