# Supplementary material for: Toward Long‐Term Reliable Human‐Machine Interaction: A Flexible, Breathable, and Self‐Powered Pressure Sensor System With Firefighting Validation
Source: Adv Sci (Weinh). 2026 May 14:e75660. Online ahead of print. doi: 10.1002/advs.75660 (PMC13335841; doi:10.1002/advs.75660)
Supplement: Supplementary file 1 — Supporting File 1: advs75660‐sup‐0001‐SuppMat.docx. [file ADVS-9999-e75660-s001.docx]

Supporting Information

Toward Long-Term Reliable Human-Machine Interaction: A Flexible, Breathable, and Self–Powered Pressure Sensor System with Firefighting Validation

Qilong Zhang, Zhao Yao*, Jiaxu Liu, Yuxuan Hou, Zhongtao Zhang*, Jingwei Xue*, Leonid Chernogor, Nam Young Kim, Eun Seong Kim*, Yuanyue Li*and Yang Li*

**
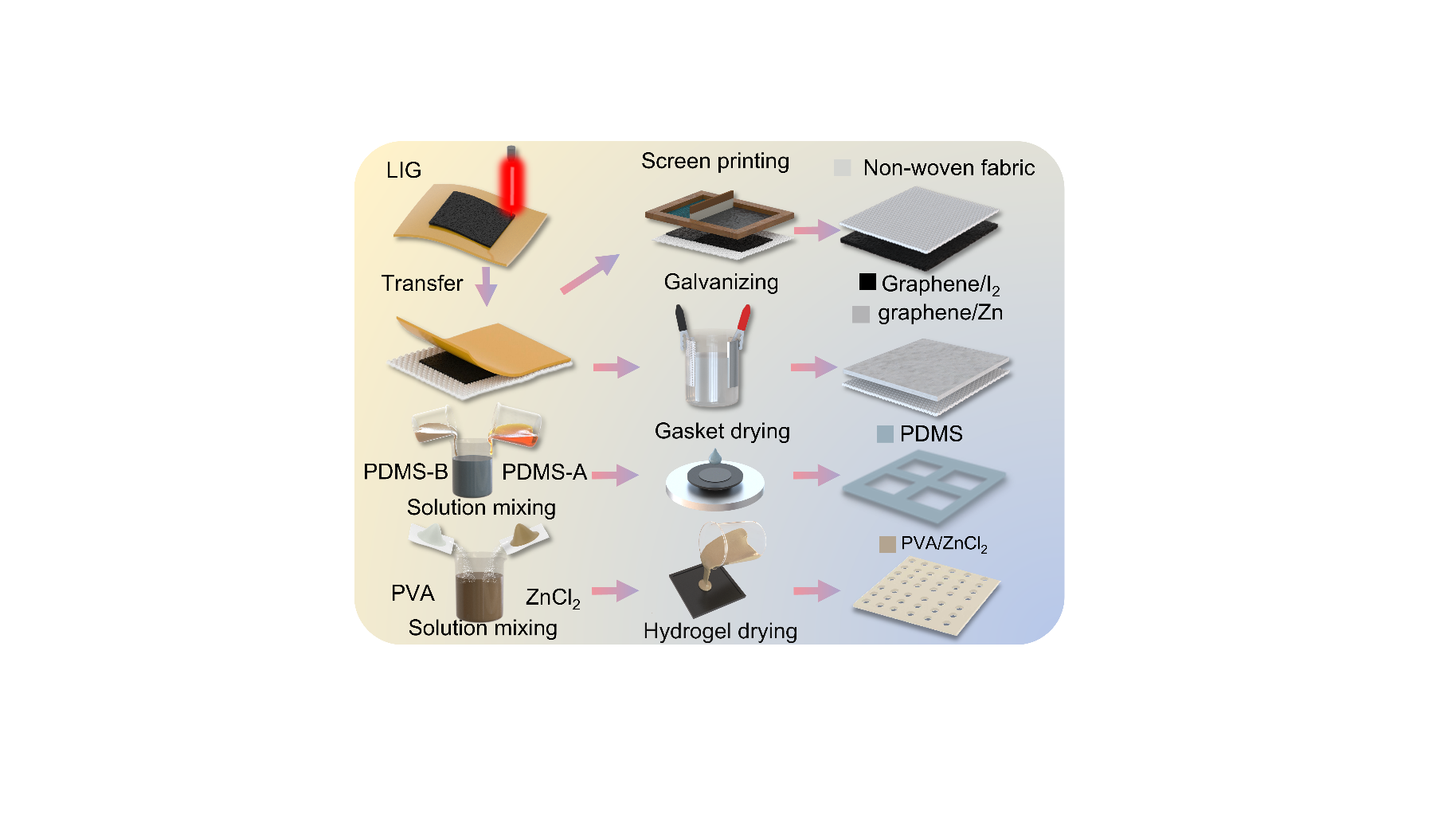
**

**Figure S1.** Manufacturing process of flexible breathable potentiometric pressure sensor based on zinc-iodine.


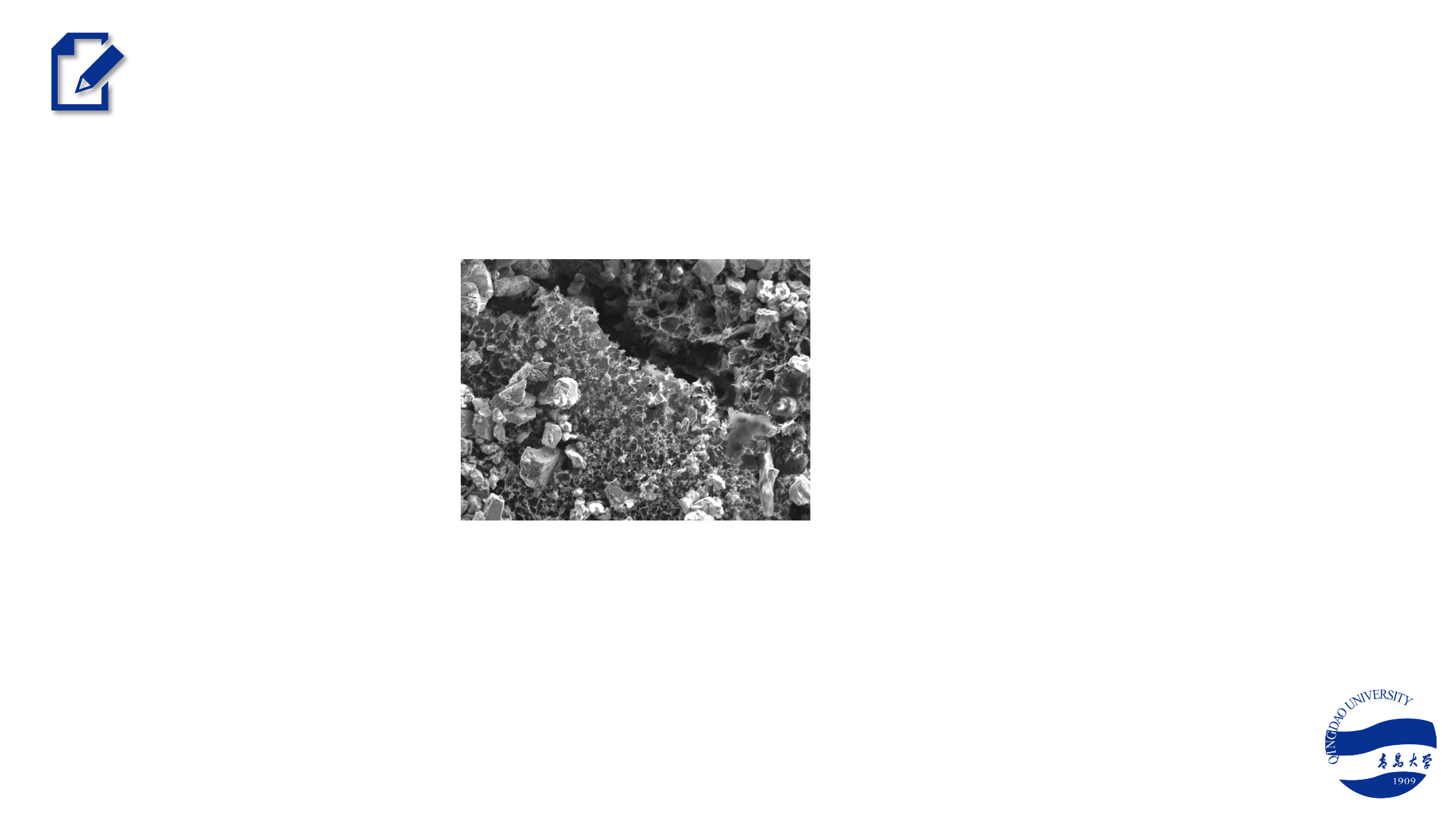


**Figure S2.** Representative SEM image of LIG generated by direct laser writing on a PI film.


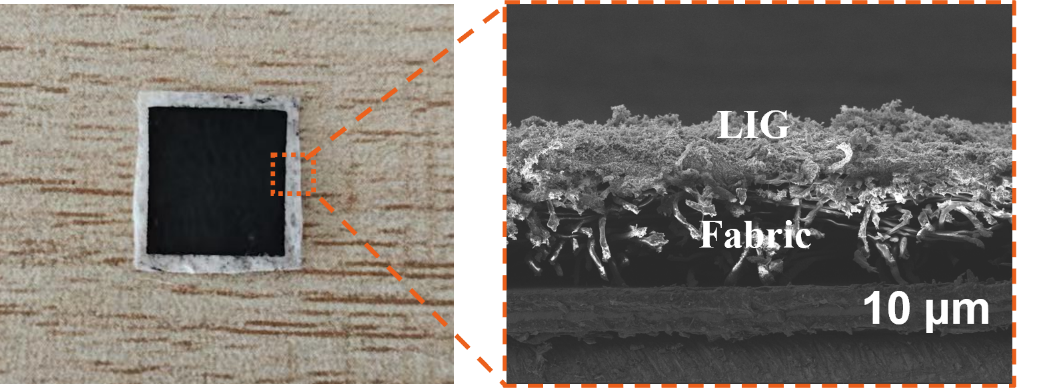


**Figure S3.** Representative SEM image of LIG relocated from a PI film onto a nonwoven fabric.


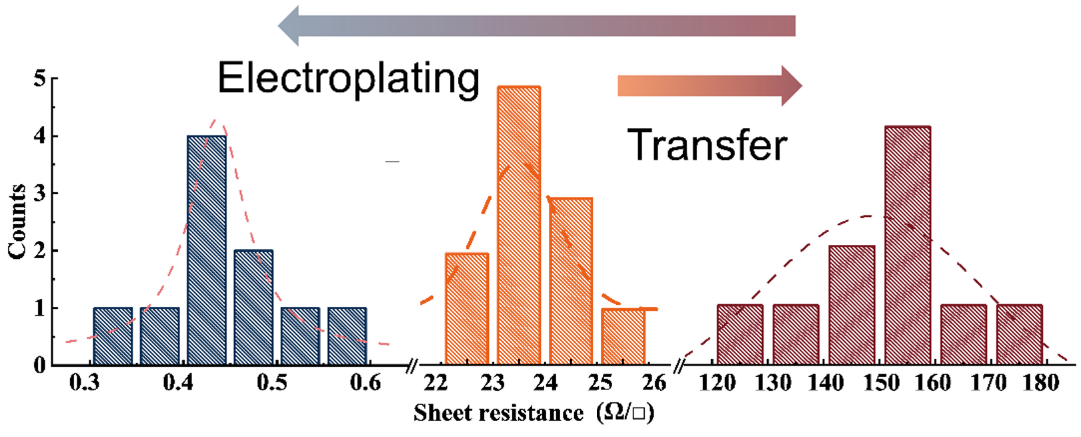


**Figure S4.** Evolution of sheet resistance during the three-stage process of LIG transfer from PI film to nonwoven fabric and subsequent zinc electroplating.

**Figure S5.** Influence of electrolyte thickness on device performance.


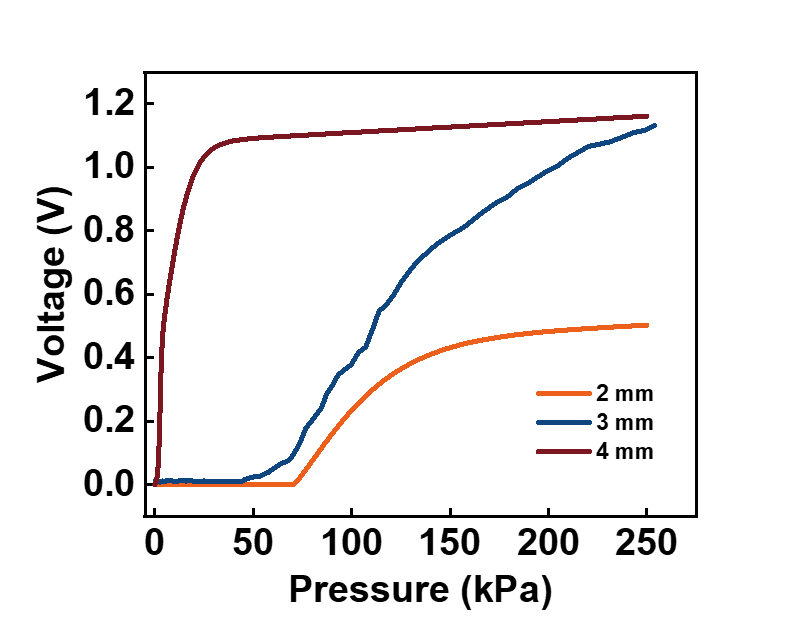


**Figure S6.** Influence of the pore size of PDMS gaskets on device performance.


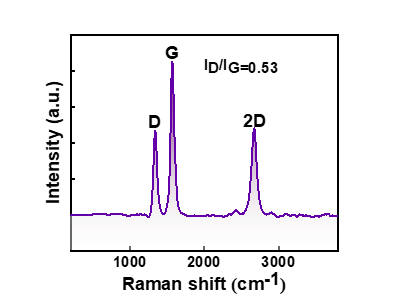


**Figure S7.** Raman spectrum of the LIG showing its structural characteristics.


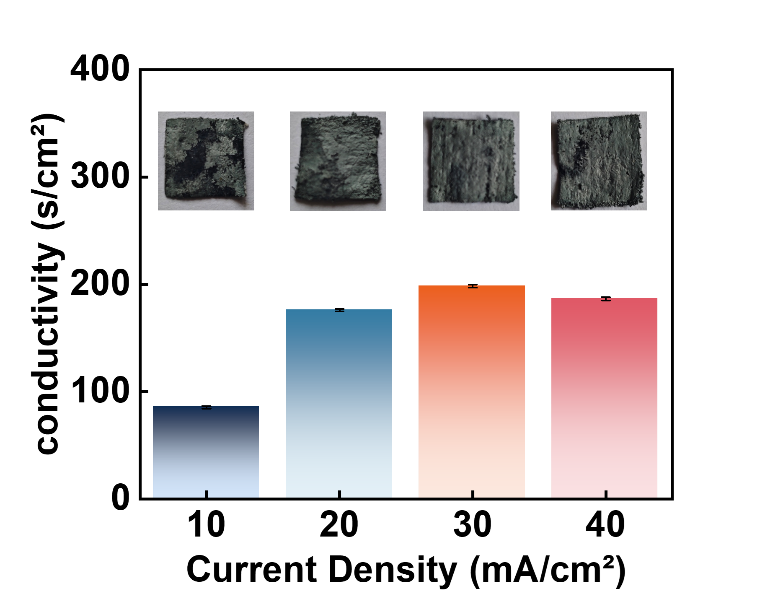


**Figure S8.** Effect of different current densities on electroplating.


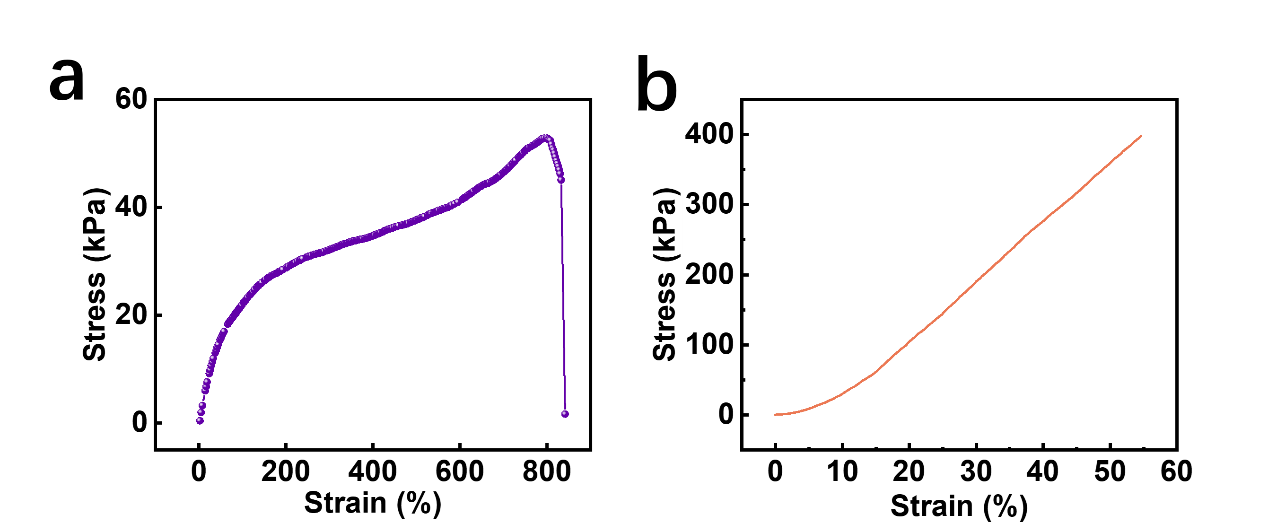


**Figure S9.** a) Tensile stress-strain curves of the PVA-ZnCl_2_ hydrogel. b) Compressive stress-strain curves of the PVA-ZnCl_2_ hydrogel.


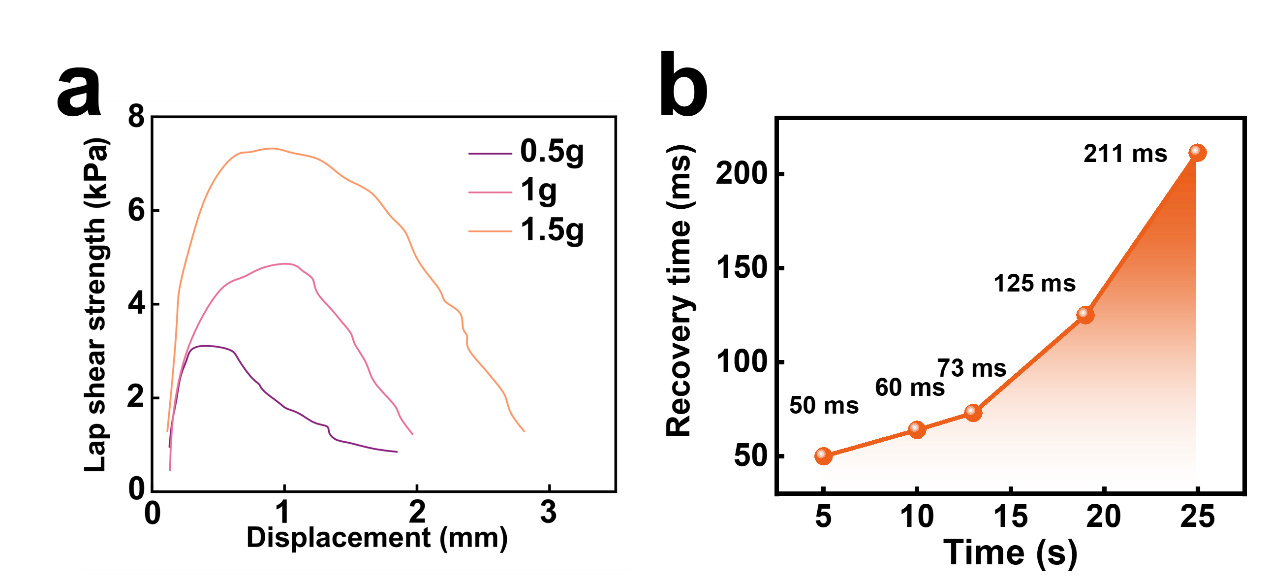


**Figure S10.** a) Lap shear adhesion strength of PVA-ZnCl_2_ hydrogels with varying ZnCl_2_ concentrations. b) Recovery time of devices with different zinc chloride concentrations.


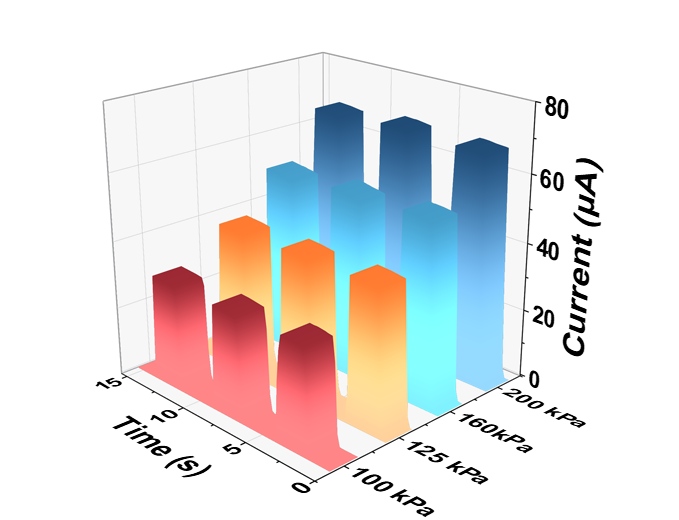


**Figure S11.** The current response of the sensor under different pressures.


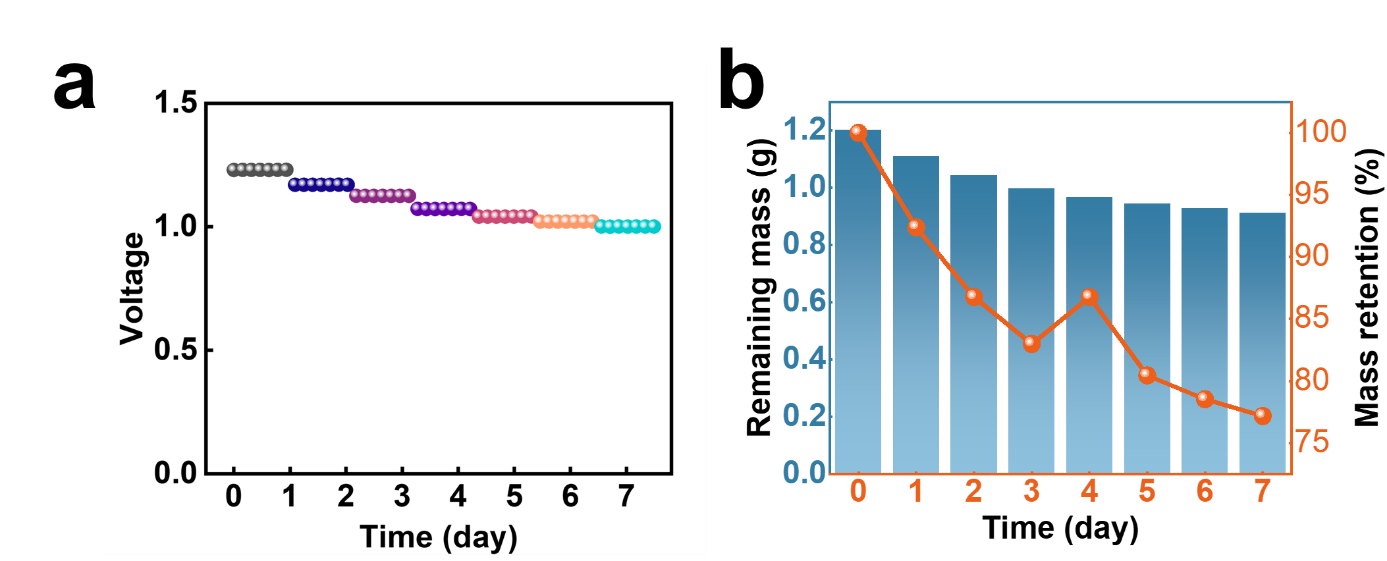


**Figure S12.** a) Operational stability of the device: output variation over one week in an ambient environment. b) Mass loss curve of the hydrogel over one week.


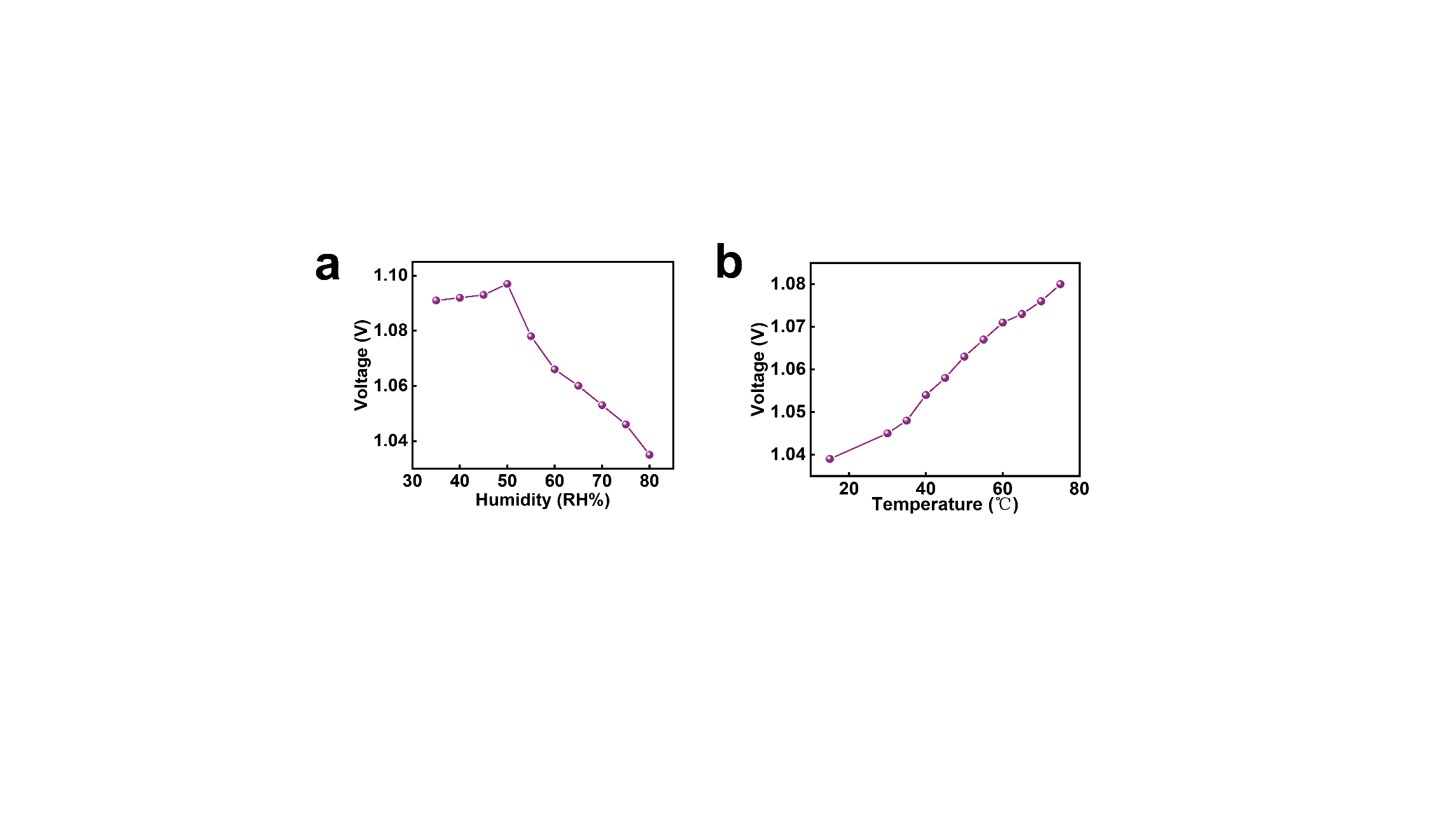


**Figure S13.** a) Sensor response under different humidity conditions. b) Sensor response under different temperature conditions.


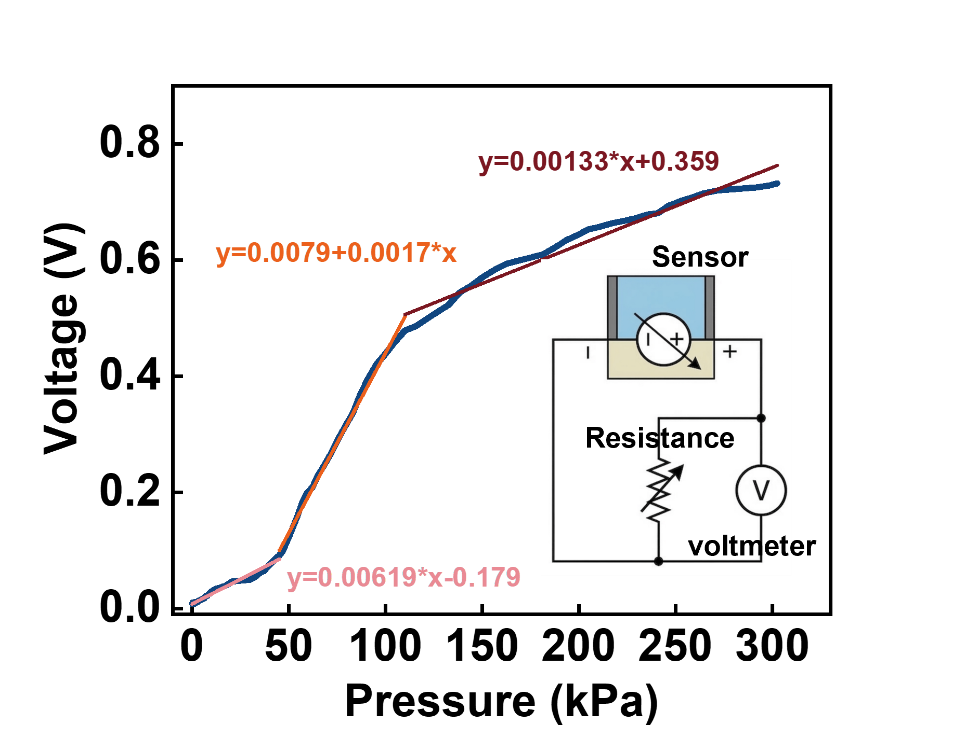


**Figure S14.** Analysis diagram of the pressure regulation mechanism of the self-powered sensor.


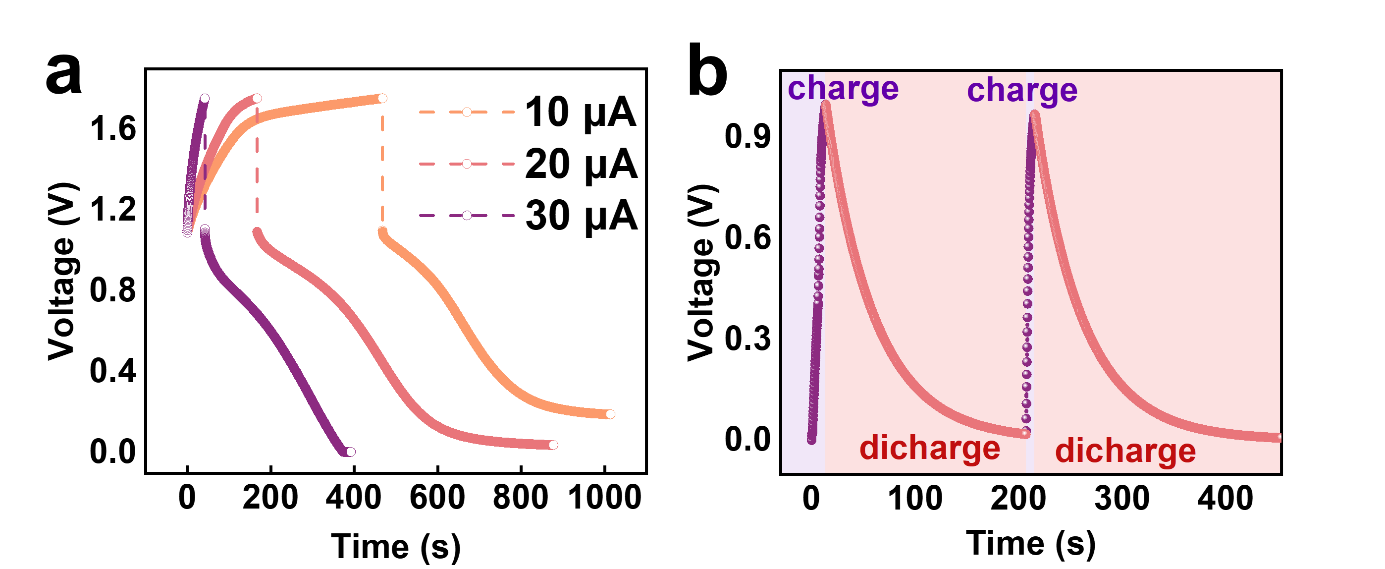


**Figure S15.** a) Galvanostatic charge/discharge profiles of the device under varying current densities. b) Two charging cycles of a capacitor by the device.


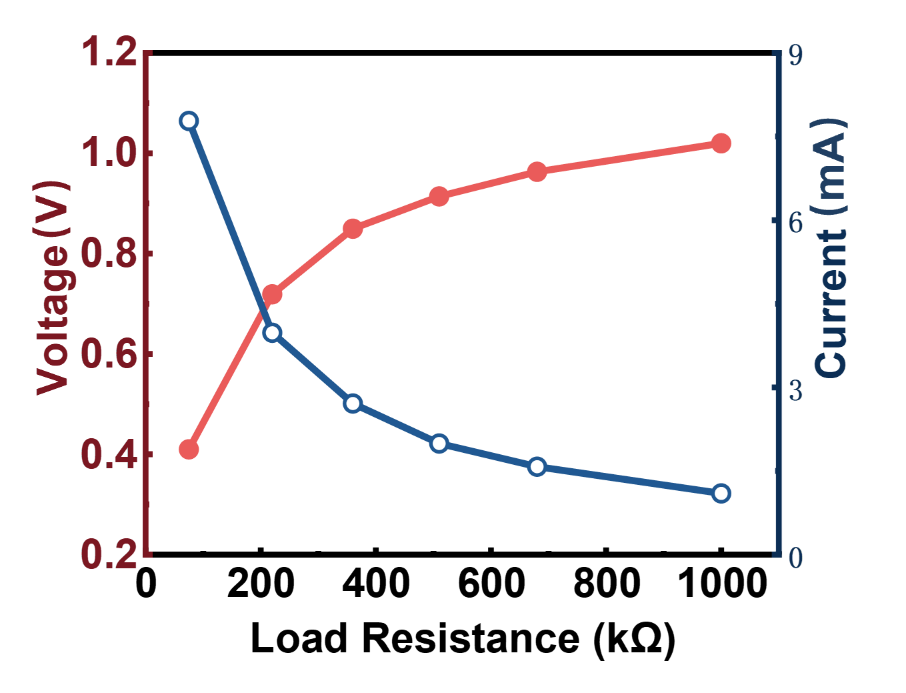


**Figure S16.** Variation of the output voltage/ current with load resistance.


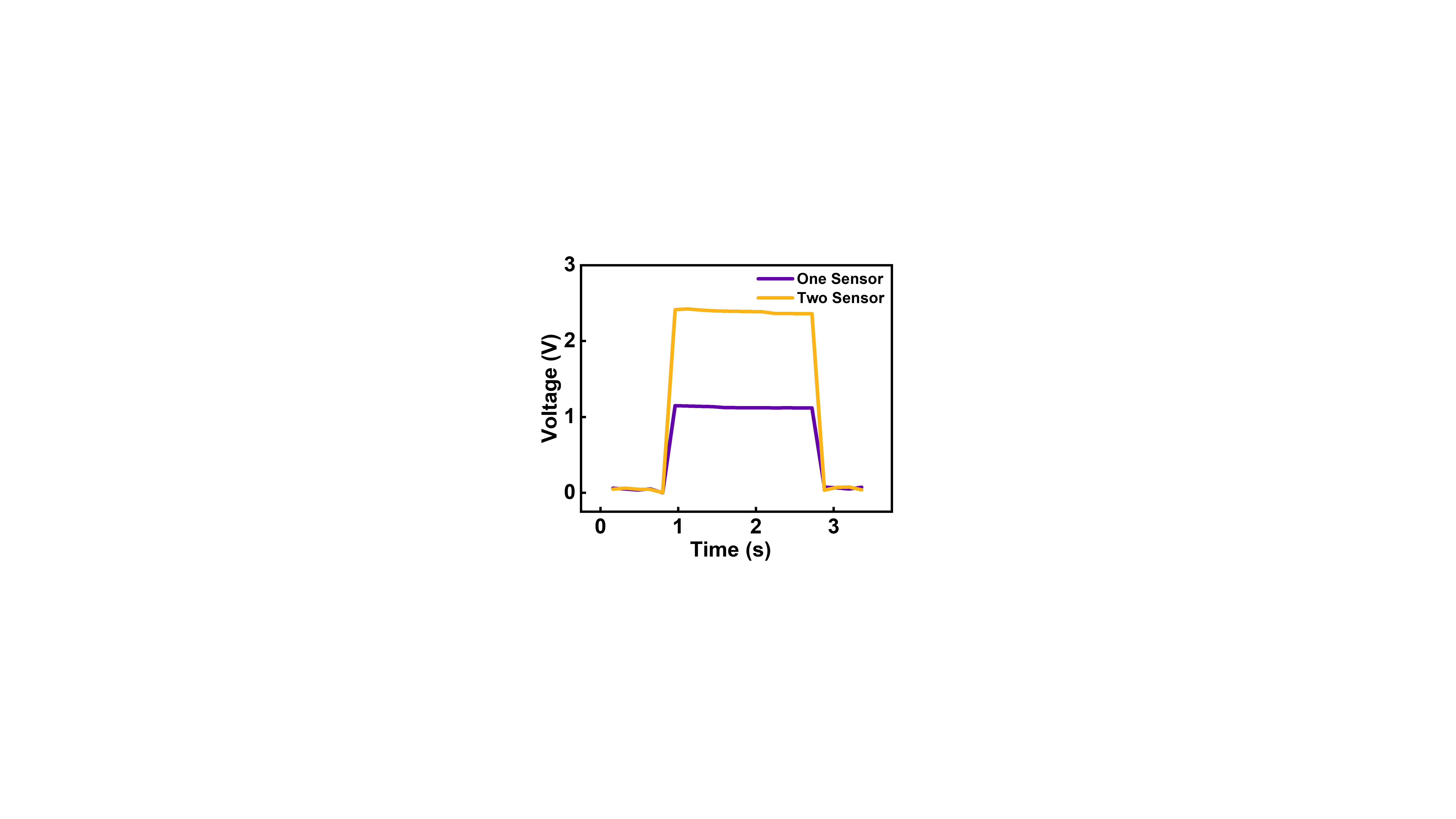


**Figure S17.** Output voltage of a single device and two devices connected in series.


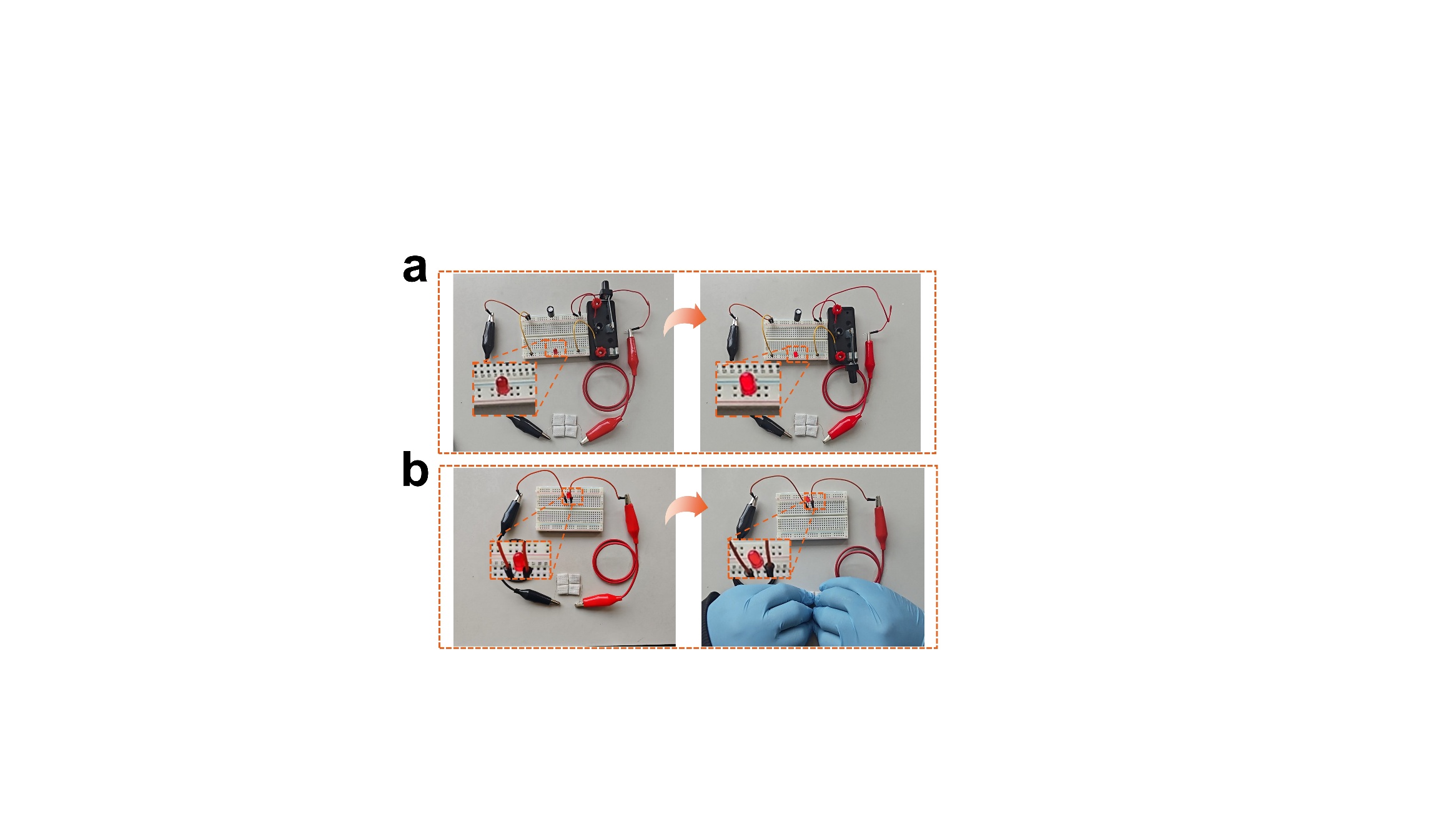


**Figure S18.** a) Capacitor charging by the sensor, discharging through LED controlled by SPDT switch. b) Demonstration of the sensor's capability to directly power an LED.


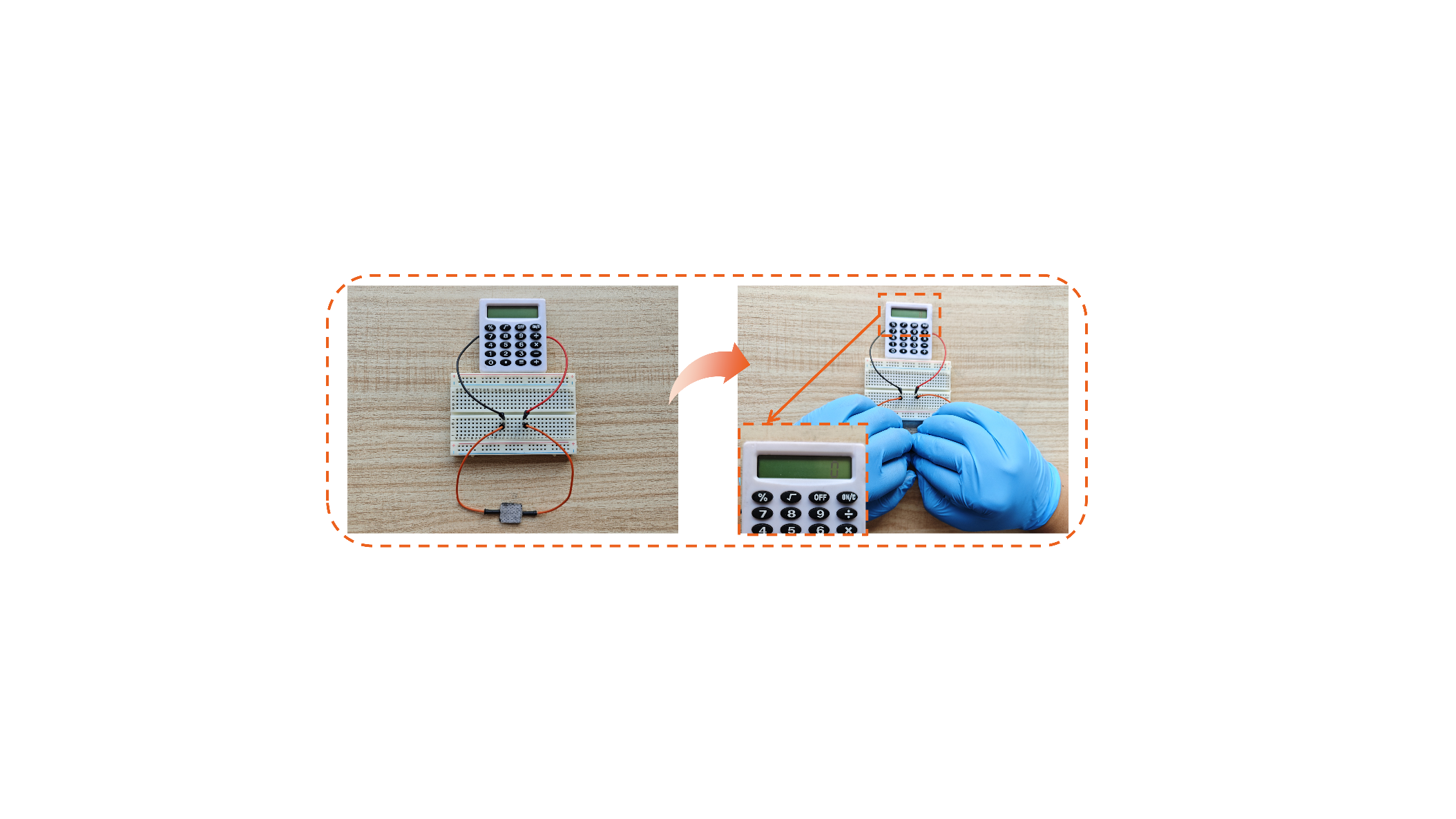


**Figure S19.** Demonstration of the sensor’s capability to power a portable calculator.


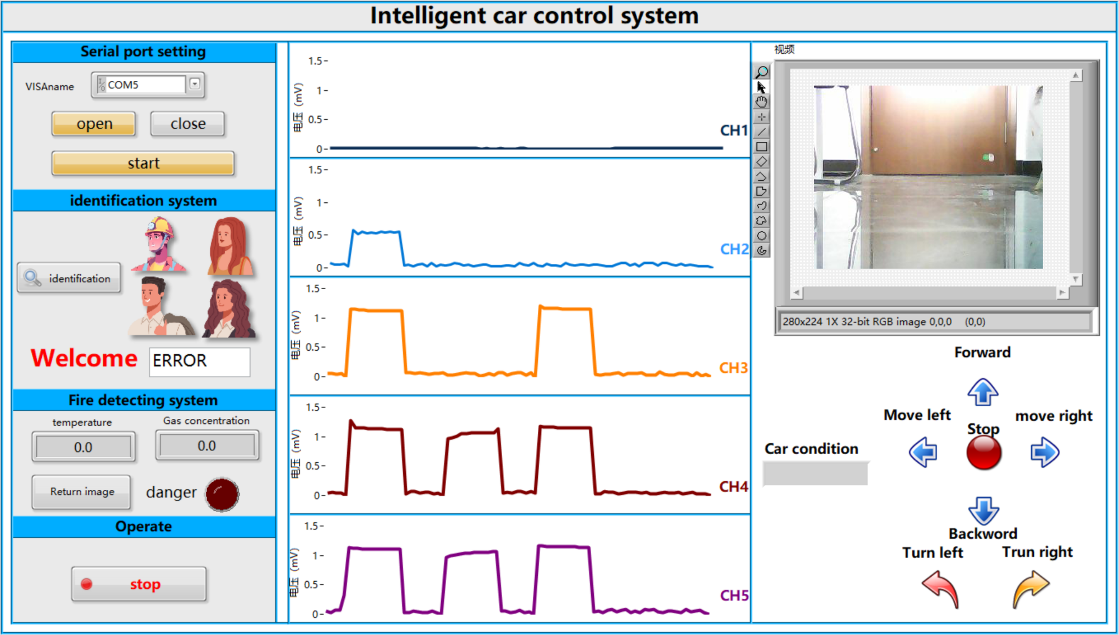


**Figure S20.** LabVIEW interface subsequent to the entry of an incorrect command.

**Video S1.** Demonstration of the sensor powering an LED.

**Video S2.** Demonstration of an LED powered by a sensor-charged capacitor.

**Video S3.** Display of the application of the user identification system.

**Video S4.** Display of the application of the intelligent fire trolley control system.
